# An Evolutionarily Young Polar Bear (*Ursus maritimus*) Endogenous Retrovirus Identified from Next Generation Sequence Data

**DOI:** 10.3390/v7112927

**Published:** 2015-11-24

**Authors:** Kyriakos Tsangaras, Jens Mayer, David E. Alquezar-Planas, Alex D. Greenwood

**Affiliations:** 1 Department of Translational Genetics, The Cyprus Institute of Neurology and Genetics, 6 International Airport Ave., 2370 Nicosia, Cyprus; 2 Department of Human Genetics, Center of Human and Molecular Biology, Medical Faculty, University of Saarland, 66421 Homburg, Germany; jens.mayer@uniklinikum-saarland.de; 3 Department of Wildlife Diseases, Leibniz Institute for Zoo and Wildlife Research Berlin, Alfred-Kowalke-Str. 17, 10315 Berlin, Germany; alquezar@izw-berlin.de; 4 Department of Veterinary Medicine, Freie Universität Berlin, Oertzenweg 19b, 14163 Berlin, Germany

**Keywords:** polar bear, Ursus, Ursidinae, retrovirus, endogenous retrovirus, next generation sequencing, genomics, phylogenetics

## Abstract

Transcriptome analysis of polar bear (*Ursus maritimus*) tissues identified sequences with similarity to Porcine Endogenous Retroviruses (PERV). Based on these sequences, four proviral copies and 15 solo long terminal repeats (LTRs) of a newly described endogenous retrovirus were characterized from the polar bear draft genome sequence. Closely related sequences were identified by PCR analysis of brown bear (*Ursus arctos*) and black bear (*Ursus americanus*) but were absent in non-Ursinae bear species. The virus was therefore designated UrsusERV. Two distinct groups of LTRs were observed including a recombinant ERV that contained one LTR belonging to each group indicating that genomic invasions by at least two UrsusERV variants have recently occurred. Age estimates based on proviral LTR divergence and conservation of integration sites among ursids suggest the viral group is only a few million years old. The youngest provirus was polar bear specific, had intact open reading frames (ORFs) and could potentially encode functional proteins. Phylogenetic analyses of UrsusERV consensus protein sequences suggest that it is part of a pig, gibbon and koala retrovirus clade. The young age estimates and lineage specificity of the virus suggests UrsusERV is a recent cross species transmission from an unknown reservoir and places the viral group among the youngest of ERVs identified in mammals.

## 1. Introduction

Retroviruses are a large and diverse family of single stranded positive sense RNA viruses [1,2]. Reverse transcription of retroviral RNA genomes into double stranded DNA (dsDNA) is a key step in the retroviral replication cycle. The resulting dsDNA genome is then integrated into the genome of the infected organism in the form of a provirus [2,3,4]. Proviral integration occasionally occurs in the germline such that viral transmission changes from horizontal (via infection) to vertical as a Mendelian trait [4]. Vertically transmitted retroviruses are designated endogenous retroviruses (ERVs) and represent the successful colonization of the host germline [5]. The result of a multitude of such colonizations is that a large percentage of vertebrate genomes are comprised of ERVs [6]. ERVs represent the remnants of ancient exogenous retroviral infections that occurred millions of years ago with their exogenous counterpart in the majority of cases being extinct [7,8,9]. Only few exceptions of current colonizations in specific ERV free-living animal populations have been observed, the koala retrovirus (KoRV) representing the most prominent example [10,11]. Unlike ERVs in other species, there are koalas in southern Australia that not only lack endogenized KoRV, they show no signs of exposure whatsoever to endogenous or exogenous KoRV. This is in contrast to other species where specific variants of an ERV may be lacking in some individuals but the ERV in general is present in all members of a species, e.g., endogenous Jaagsiekte sheep retrovirus (enJSRV), in sheep which are polymporphic or human endogenous retrovirus type K, human mouse mammary tumor virus like subgroup 2 elements (HERV-K (HML-2)) in humans, some of which are also polymorphic [11,12,13,14]. Over time, ERVs may accumulate mutations, deletions and insertions that render many of them inactive and non-pathogenic, with only a small fraction or none being able to produce functional retroviruses [7,15,16]. The minority of ERVs that remain active have been linked to both protection of the host from exogenous viruses and with a variety of diseases such as tumors, neurodegenerative diseases, and immunodeficiencies [17,18].

The best-characterized ERV groups are those of humans and laboratory mice with most other species either poorly investigated or not at all. The investigation of wildlife has revealed exceptional genetic diversity and evidence of cross species transmission among a wide variety of taxa particularly among bats and rodents [19]. As genome data from a growing number of species becomes publicly available, a greater diversity of ERVs and their origins and evolution can be characterized. Whole genome next generation sequencing (NGS) and assembly of several mammalian genomes recently made possible the characterization of novel endogenous retroviruses in the *Ursidae*, *Megachiroptera,* and *Microchiroptera* [5,20,21,22,23].

The family *Ursidae* is comprised of three extant genera including the (i) *Ailuropoda*, containing the only non-carnivorous bear species, the giant panda (*Ailuropoda melanoleuka*); (ii) *Tremarctos*, with the spectacled bear (*Tremactos ornatus*), the only extant species; and the (iii) *Ursinae* that is represented by six extant species. Nuclear and mitochondrial DNA analysis indicates that the giant panda represents a basal lineage with a subsequent branching of the *Tremarctos* lineage. The *Ursinae* lineage, which is considered the youngest, branched from a common ancestor of the *Tremarctos* lineage [24]. Divergence of the giant panda and spectacled bear lineages is estimated to have occurred 20 million years ago, whereas the *Ursinae* originated in the past five to eight million years [25,26]. Recently, genomic data has been made publicly available for several bear species providing the necessary resources for characterization of their ERV content [27,28,29].

Analysis of two polar bear viromes identified endogenous viral sequences with similarity to Porcine Endogenous Retrovirus (PERV), a gammaretrovirus that is present in all porcines [30]. In the current study, using the polar bear draft genome and whole genome sequencing data from various bear species, including a sub-fossil polar bear from the Poolepynten coastal cliff in Svalbard [31], we characterized a endogenous gammaretrovirus, which we designate UrsusERV, with regards to its evolutionary age, species distribution, and phylogenetic relationship among other gammaretroviruses. The results suggest that along with koalas and sheep, polar bears may carry very recently endogenized retroviruses.

## 2. Results

### 2.1. Identification of an Evolutionarily Young Gammaretroviral ERV in Polar Bears

A virome study examining the brain tissue of two polar bears (Knut of the Berlin Zoological Garden (Berlin, Germany) and Jerka of the Wuppertal Zoological Garden (Wuppertal, Germany) identified sequences ranging from 73% to 80% identity, at the DNA level, to porcine endogenous retrovirus (PERV), *Mus dunni* endogenous retrovirus (MDEV), Feline sarcoma virus (FeLV), and Murine leukemia virus (MLV) genes [23].

A blastn search was performed on all 72,214 scaffolds of the draft polar bear genome sequence using, as query sequences, brain derived transcriptome retroviral sequences with the highest percentage similarities to MDEV and PERV [21]. MDEV and PERV sequences attracted the most non-overlapping transcriptome reads, deriving from different parts of the retroviral genome, from the RNA-seq virome data and were therefore further analyzed [23]. The blastn search revealed four loci in four different polar bear scaffolds with significant similarity to the potential retroviral sequences (Table S1). Presumed endogenous retrovirus harboring loci were extracted from the respective polar bear scaffold sequences including ~15,000 bp both up and downstream of the blastn identified scaffold regions. The exact boundaries of the identified ERV loci were then determined using Repeatmasker (Table S2), and additional sequence comparisons, as described in [32,33,34]. Repeatmasker results indicated that the identified proviruses belonged to the gammaretrovirus genus of retroviruses and were annotated as koala retrovirus (KoRV) sequences indicating the high similarity to KoRV, specifically KoRV_I-int (Table S2) [35,36].

**Figure 1:**
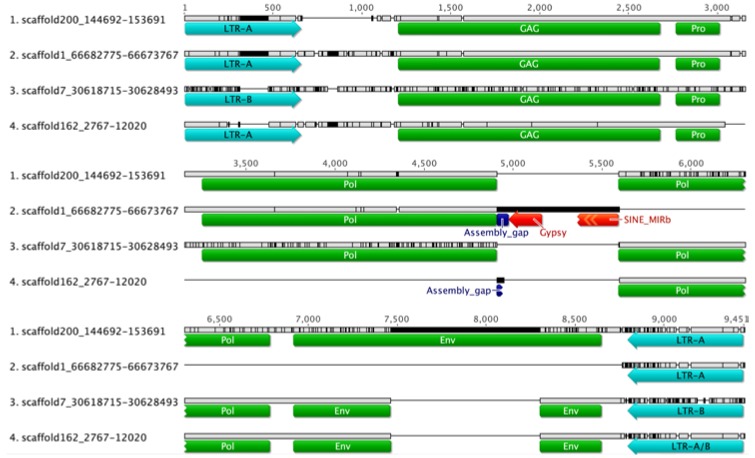
Multiple sequence alignments of the four UrsusERV proviral loci as identified in the polar bear genome sequence are shown. Proviral long terminal repeat (LTR), *gag*, *pro*, *pol* and *env* regions are indicated. Scaffold 1 and scaffold 162 proviral sequences harbor assembly gaps and lack proviral *pol* and *env* regions, indicated by horizontal black lines. The scaffold 200 provirus is structurally complete and could potentially produce functional proteins. Sequence differences relative to the consensus sequence are indicated by black vertical lines with the scaffold 7 provirus exhibiting the greatest overall divergence. Only the scaffold 200 provirus harbors a complete *env* gene that is partially present in scaffolds 162 and scaffold 7 and completely absent from scaffold 1.

The Retrotector script was used as an independent verification method for the presence of retroviral motifs in the polar bear scaffolds. Retrotector analysis revealed retroviral motifs similar to known gammaretroviral sequences [33,34,37] identifying proviral genes *gag*, protease (*pro*), polymerase (*pol*), and envelope (*env*) as shown in Figure 1. A dUTPase domain was not identified. Retrotector generated reconstructed retroviral proteins (called putein) sequences from the defective reading frames of the proviral loci by introducing 10 frameshifts. GAG and PRO proteins were predicted from Retrotector for all four scaffolds with a percentage identity of 94.8% and 80%, respectively. The majority of the differences found in the alignments were identified in scaffold 7 that also contain three frameshifts in the GAG putein (Figure 1). Excluding scaffold 7 sequences, the percentage sequence identity increased to 99% for both alignments. Putein sequences for the *pol* genes of scaffold 1 and 162 were generated from provirus regions harboring assembly gaps and were therefore incomplete, while proviral sequences within scaffold 7 and scaffold 200 appeared to be intact with regard to the *pol* gene region with a percentage identity of 95.6% (Figure 1). Retrotector analysis corrected 5 frameshifts during the scaffold 1 POL putein prediction, further indicating the incompleteness of the identified ORF. The *pol* gene in scaffold 1 was interrupted by an inserted Gypsy like element and a SINE-MIRb element encompassing nucleotides 4652–5549 of the provirus.

Motifs conserved in retroviral envelope proteins were detected by Retrotector in all proviral sequences, with the exception of the scaffold 1 proviral sequence that was lacking the *env* gene region. The *env* gene putein domains in scaffolds 7 and 162 appeared to be affected by mutations. Generation of puteins for both scaffolds identified frameshifts, whereas the provirus in scaffold 200 displayed an intact ENV ORF (Figure 1). Envelope puteins, as generated by Retrotector, displayed 91.8% among sequence identity for alignable regions.

The National Center for Biotechnology Information (NCBI) Conserved Domain database analysis of proviral scaffolds identified the same motifs and frameshifts as Retrotector for all four proviral sequences and further highlighted the degree of similarity with respect to conserved motifs in other retroviral proteins [38]. Further analyses of all proviral loci indicated tRNA^Pro^ as the primer binding site for viral replication initiation. Sequence analysis further indicated that proviral loci in scaffold 1, 7 and 162 are unlikely to produce functional viruses due to frameshifts, deletions, insertions, and repetitive elements that interrupt the sequences. The provirus in scaffold 200, on the other hand, harbored intact retroviral motifs in the correct reading frame, an indication that functional viral proteins could potentially be produced from this locus (Figure 1).

**Figure 2:**
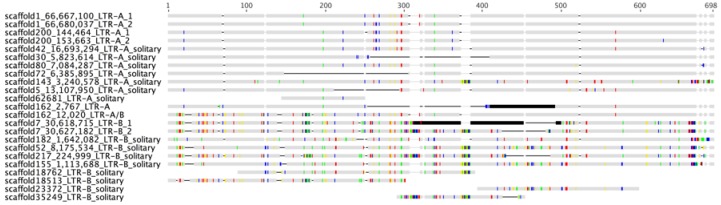
Multiple sequence alignments of UrsusERV LTRs, as identified in the polar bear genome sequence, are shown. LTR-A represents the first group of LTRs while LTR-B represents the second. Proviral 5′ LTRs and 3′ LTRs are indicated by numbers 1 and 2, respectively, in the sequence names. Solitary LTRs are indicated accordingly. Some identified LTRs only comprised sub-regions of the full-length LTR. Colored vertical lines represent nucleotide differences among the LTR sequences with respect to a majority rule consensus sequence (C: blue; G: yellow; A: red; T: green). Horizontal lines indicate gaps in the alignment while dark grey shaded regions indicate assembly gaps in some of the LTR sequences. The 3′ LTR in the scaffold 162 provirus is a recombinant between an LTR-A and LTR-B type LTR.

A majority rule proviral consensus sequence of 7878 nt was generated from the four identified proviral loci. A consensus sequence that included LTR, *gag*, *pro*, *pol*, and partial *env* gene characteristics was used for additional blastn searches of the polar bear draft genome sequence scaffolds. Blastn search using the consensus sequence identified no additional proviral loci. However, 15 solitary LTRs were identified. A multiple sequence alignment of identified UrsusERV LTR sequences (proviral and solitary) revealed two subgroups with group-specific nucleotide differences that we designated LTR-A and LTR-B (Figure 2). Out of a total of 23 LTRs, 12 belonged to the LTR-A subgroup, 10 belonged to the LTR-B subgroup and one appeared to be a recombinant. The scaffold 1 and 200 proviruses were flanked by LTR-A and the scaffold 7 provirus was flanked by LTR-B.

We observed that the scaffold 162 provirus was flanked by a 5′ LTR characteristic of an LTR-A sequence, yet the 3′ LTR was characteristic of an LTR-B sequence for the 5′ most 200 nt and an LTR-A-like sequence for the 3′ most 300 nt. UrsusERV LTR sequences were therefore analyzed for recombination events using four computational methods, specifically, GARD, DualBrothers, Recombination Analysis Tool (RAT, Norwich, UK) and RECCO (Saarbrücken, Germany) [39,40,41,42,43]. All four methods identified a recombination event for the 3′ LTR in the scaffold 162 provirus occurring between nt 177 and 207 of the 3′ LTR sequence (Figure S1). Phylogenetic analysis of the GARD recombination script clustered the first sub-region of scaffold 162 LTR-A/B ranging from 1 to 177 bp within the LTR-B clade, while the second region ranging from 207 bp onwards within the LTR-A clade, supporting a recombination event (Figure S1). The RECCO computational method confirmed the presumably recombined LTR sequence (“scaffold162_1-18,384_LTR-A/B” in Figure S1) with a *p*-value < 0.001. RECCO also indicated another potential recombination event in the Scaffold 143 LTR (Table 1).

**Table 1:** RECCO analysis identified potential recombination events in two of the UrsusERV LTRs *.

Sequence	Start	End	Savings	Seq pv
scaffold143_3,240,578-3,261,249_LTR-A_solitary	311	311	8	0,000999
scaffold162_1-18,384_LTR-A/B	186	216	30	0,000999

* Sequences with identified recombination events are shown in the left column, followed by start and end positions of the region in which the recombination event likely occurred. “Savings” illustrate the mutational cost saved due to the recombination event. Sequence p-value (Seq pv) gives the likelihood of a recombination having occurred.

Target site duplications flanking proviral LTRs were also examined. Retroviral integration generally generates a target site duplication (TSD) of 4–5 bp immediately flanking the provirus [44]. Four bp TSDs were identified for the four proviral insertions and in 10 out of 15 solo LTRs (Table 2). The remaining five LTR sequences were identified in small scaffold sequences with TSD information being unavailable in the current draft genome version.

**Table 2:** UrsusERV LTR and solitary LTR 4 bp target site duplication (TSD) sequences.

Polar Bear Scaffolds	TSD Sequence
Scaffold 1	CATC/CATC
Scaffold 7	ACTT/ACTT
Scaffold 162	AGGC/AGGC
Scaffold 200	ATGG/ATGG
Scaffold 182	CTTC/CTTC
Scaffold 30	CTCT/CTCT
Scaffold 80	GTGT/GTGT
Scaffold 42	ATCT/ATCT
Scaffold 52	TATC/TATC
Scaffold 217	GTAG/GTAG
Scaffold 143	TGAA/TGAA
Scaffold 72	GCAC/GCAC
Scaffold 155	AGAC/AGAC
Scaffold 5	GTAT/GTAT
Scaffold 18,513	N/A
Scaffold 18,762	N/A
Scaffold 23,372	N/A
Scaffold 35,249	N/A
Scaffold 62,681	N/A

N/A: TSDs could not be determined for the corresponding loci (see the manuscript text).

### 2.2. UrsusERV Age Estimation

Age estimation was based on the sequence divergence of the proviral 5′ and 3′ LTR sequences. Once the provirus is integrated into the genome of the host, the 5′and 3′ LTRs are identical in sequence [37,38]. Accumulation of differences between the proviral 5′ and 3′ LTR will subsequently occur at the mutational rate of the host genome. Thus, sequence divergence between the proviral 5′ and 3′ LTRs can be used to estimate the age of a given provirus [45]. Using an evolutionary rate of 0.0015/nt/myr, as previously determined for bears [25], provided integration age estimations of up to two million years old with the provirus in scaffold 200 lacking differences between its two LTR sequences making an age estimate based on a molecular clock difficult.

The oldest provirus located in scaffold 7 was estimated to be roughly 2 myr old (Table 3). The scaffold 162 provirus could not be dated with confidence, as the 3′ LTR is a recombinant between LTR-A and LTR-B types. Proviral distribution analysis of UrsusERV in ancient polar bear whole genome sequence data (130,000 year old fossil) identified sequence reads spanning the Scaffold 200 LTR ends and flanking regions. Presence of the youngest provirus in the ancient polar bear genome suggests it integrated in the polar bear lineage at least 130,000 thousand years ago and likely earlier [31]. We stress in this context that age estimations based on LTR divergence should be regarded with caution, especially when regarding evolutionarily younger proviruses, as this dating method can be biased by, for instance, gene conversion thus potentially underestimating proviral ages [46,47].

**Table 3:** UrsusERV provirus age estimations using LTR divergences *.

Polar Bear Scaffolds	5′ LTR Location	3′ LTR Location	Overall Mean K2P Distance	Age Estimation Mya
Scaffold 1	66,674,296–66,674,797	66,681,308–66,681,790	0.002	0.625
Scaffold 7	30,626,500–30,627,187	30,633,692–30,634,278	0.007	2.1875
Scaffold 162	7152–7643	13,824–14,441	ND	ND
Scaffold 200	145,219–145,715	152,850–153,350	0	0

* Proviral age estimates are given as millions of years ago (Mya). Proviral ages were calculated based on Kimura-2-parameter (K2P) corrected sequence divergence between proviral 5′ and 3′ LTRs (see main text). Location of proviral 5′ and 3′ LTRs is given for each scaffold. Proviral age estimates are given as millions of years. An age for Scaffold 162 could not be determined with certainty due to a recombination event in the 3′ LTR between an LTR-A and B type LTR. The Scaffold 200 provirus had identical LTRs making age estimates difficult but suggest it is an evolutionarily young integration. Age estimates not determined are indicated by ND.

### 2.3. UrsusERV Molecular and Genome Screening of Bear Species

We further investigated the presence of UrsusERV sequences in additional polar bears and other bear species. Majority rule consensus sequences of proviral and LTR sequences were used to screen the giant panda draft genome assembly (BGI-Shenzhen AilMel 1.0 December 2009) available through the University of California, Santa Cruz (UCSC) Genome Browser [48]. Blat searches failed to identify significantly similar sequences indicating that UrsusERV is not present in all members of the Ursidae family, further supporting the age estimations based on LTR sequence divergence. We next examined other bear species, specifically whole genome sequencing data from black bear (*Ursus americanus*), brown bear (*Ursus arctos*), grizzly bear (*Ursus arctos ssp.*), an ancient polar bear jawbone [28,31], and six unrelated modern polar bears that were obtained from the National Centre for Biotechnology Information (NCBI) Sequence Read Archive [27]. Short read data were transformed to fastq files and mapped to the four polar bear retroviral scaffolds using the Burrows–Wheeler aligner [49]. Analyses of the polar bear mapped reads indicated the presence of all four proviruses in the six polar bear whole genome sequenced animals. Reads identical to UrsusERV proviral sequence were also identified in the ancient polar bear jawbone sequence data. Genome sequence data from the black and brown bears identified sequences with 93.9% identity to the identified consensus proviral sequence in both bear species indicating that UrsusERV is present in the *Ursinae* clade of the *Ursidae* family [50]. This also suggests integration of UrsusERV like viruses in bears occurred earlier than 2 million years ago (mya, the oldest dated provirus) as the black bear and brown/polar bear clades diverged from a common ancestor over 7 mya.

All the UrsusERV sequences flanking the polar bear proviral LTRs in the polar bear draft genome sequence were identified in the other six polar bear whole genome draft sequences. The scaffold 200 3′ proviral insertion site was the only one identified in the 130,000-year old polar bear jawbone sample providing evidence that the same insertion site for at least one provirus was present in the ancient polar bear population. Sequence coverage was not as comprehensive as for the modern bear sequences and thus we cannot exclude that the other proviruses were present in this sample [31]. Brown and black bears whole genome draft sequences were also screened for the proviral insertion sites identified in polar bears. While the proviral flanking sequences could be identified in the black bear genome, they lacked a proviral integration e.g., the flanking sequences were uninterrupted by UrsusERV. Therefore, while black bears are positive for UrsusERV sequences, the virus has integrated in distinct locations in the genome relative to polar bears. This could in part explain the differences in age estimates between the polar bear derived ERVs and the divergence time of black bear and brown bear/polar bear lineages in addition to the overall lower percentage identity of UrsusERV derived from black bears relative to those found in brown or polar bears. Out of the four female brown bears examined, two had the same UrsusERV insertion sites as polar bears for all the proviruses except for the provirus located in scaffold 200, which was polar bear specific as all brown bears tested lacked an integration at this genomic location although the flanking sequences were identified (Table S3). Two additional brown bear genome sequences, from Kenai and Admiralty Island, where found to have the same provirus insertions found on polar bear scaffolds 7 and 162, while the polar bear scaffold 1 integration was absent indicating insertion site polymorphisms in brown bears for the scaffold 1 proviral integration (Table S3) [51].

**Figure 3:**
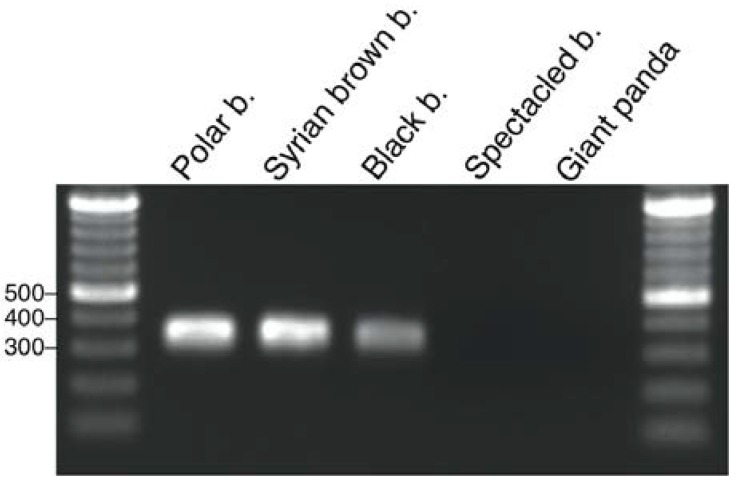
Gel electrophoretic separation of PCR products amplified from five bear species. PCR products and a 100 bp DNA ladder were separated through a 1.5% agarose gel. As indicated by a PCR product of an expected size of 312 bp, UrsusERV sequences were present in the examined polar, brown and black bears but absent from spectacled bears and the giant panda. Relevant sized DNA marker bands are indicated on the left.

Molecular screening for UrsusERV sequences was also performed by sequence-specific PCR using genomic DNA extracted from a giant panda, spectacled bear (*Tremarctos ornatus*), Syrian brown bear (*Ursus arctos syriacus*), black bear, and polar bear to further verify the bioinformatic analysis. Genomic DNA was subjected to PCRs using primers designed to amplify a 312 bp region within the *gag* gene region showing little sequence divergence between three bioinformatically identified UrsusERV proviruses. PCR products of expected sizes were amplified from polar bear, Syrian brown bear and black bear, but not from spectacled bear and giant panda (Figure 3). Sanger sequencing of the PCR products confirmed that the target *gag* region of UrsusERV was amplified (Figure S2). This approach further demonstrates that UrsusERV is absent from the panda and spectacled bear genomes but present in all examined members of the *Ursinae* subfamily of bears.

### 2.4. UrsusERV Phylogenetic Analysis

**Figure 4:**
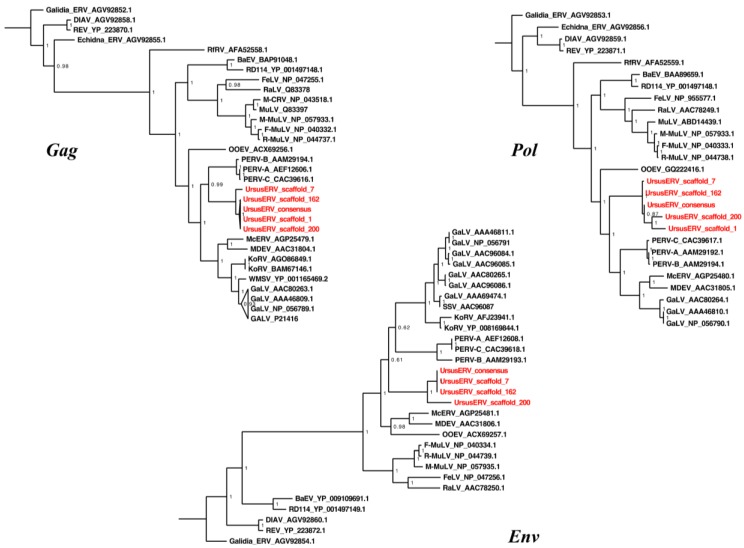
Phylogenetic analysis of consensus and individual UrsusERV proviral proteins within the *Retroviridae*. Bayesian phylogenetic trees are shown for GAG, POL, and ENV proteins. Protease analysis due to limited variation among the sequences was included with the polymerase analysis. Posterior probabilities >50% are shown. All sequences and analysis description are included in the material and methods section. UrsusERV (highlighted red) consensus and proviral sequences form a distinct clade that in all three analyses is closely related to PERV sequences.

**Figure 5:**
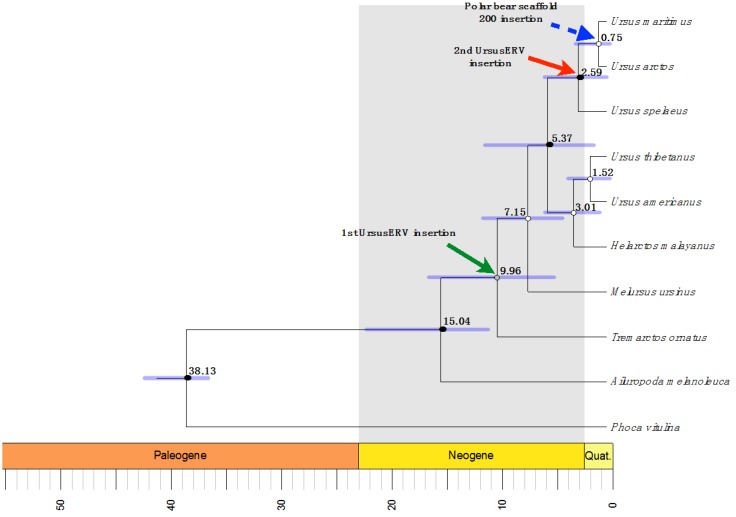
Maximum clade probability tree from mtDNA and nuclear DNA alignment display the Beast chronogram estimation. Clades age was estimated using fossil data points, with log normal distribution, and GTR model. Median divergences ages are shown above the blue horizontal bars that indicate the 95% highest posterior intervals of the estimation. Green arrow indicates the UrsusERV first insertion into the Ursinae clade that affected most likely all members of the clade. Red arrow indicates the second UrsusERV insertion into the brown bear clade, while the blue arrow represents scaffold 200 provirus that appears to be polar bear specific.

We next examined phylogenetic relationships of UrsusERV with other retroviruses. Protein consensus sequences were generated for the GAG, POL, and ENV genes using the Retrotector putein predictions. The protein consensus sequences were searched against the NCBI protein database and sequences with identity >35% were extracted. The UrsusERV consensus, individual scaffold protein sequences, and protein sequences extracted from NCBI protein database were aligned using MAFFT [52]. Resulting protein alignments included murine, primate, koala, bat, avian, pig, and feline gammaretroviruses. Galidia ERV protein sequences, a basal class 1 gammaretroviral group, were used as an outgroup to perform Bayesian phylogenetic analysis (Figure 4) [6]. GAG and POL trees displayed almost identical topologies with UrsusERV and the gibbon ape, koala, swine, bat, killer and whale retroviruses forming a clade sister to the murine and feline retroviruses clade. UrsusERV demonstrated greatest affinity with the PERVs for *gag* and was a sister clade to PERVs, gibbon ape leukemia viruses (GALVs), KoRVs and *Mus caroli* endogenous retrovirs (McERV)/*Mus dunni* endogenous retrovirus (MDERV) for *pol* and *env* (Figure 4). Among the individual proviruses, the scaffold 1 and 200 sequences representing the two youngest viral integrations occupied derived positions in the GAG and POL puteins trees. The ERV with the oldest integration date (scaffold 7) and the recombinant ERV on scaffold 162 occupied basal positions. This was not the case for the ENV putein tree where most scaffold sequence branching patterns could not be resolved and the youngest ERV (scaffold 200) occupied a basal position in the UrsusERV clade. However, with the exception of scaffold 200, much or all of the ENV protein was absent complicating phylogenetic analysis. Black and brown bear UrsusERV consensus sequence were constructed using the polar bear UrsusERV consensus sequence as reference. Phylogenetic analysis based on nucleotide sequences was also performed, where alignable, the black bear UrsusERV sequence was basal to the UrsusERV clade, while the brown bear UrsusERV sequence formed a sister clade to the polar bear sequence. Comparison of the UrsusERV clade with the other retroviral nucleotide sequences produced identical results to tree topologies from protein sequences. (Figures S3 and S4). The relative ages of bear lineages with respect to estimated bear lineage age, geological epoch and UrsusERV invasion events are shown in Figure 5.

**Figure 6:**
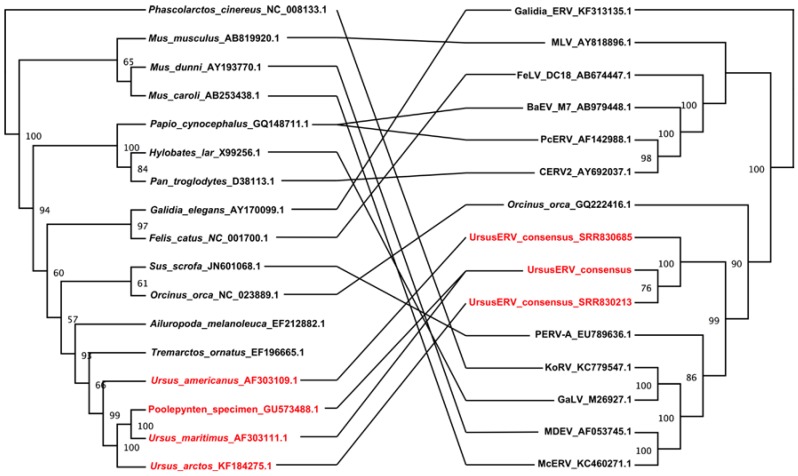
Tanglegram illustration of the phylogenies of gammaretroviruses and their hosts. The host tree on the left was based on mtDNA *cytochrome b* gene sequences, and the retroviral tree on the right was based on the polymerase gene nucleotide sequence. Ursinae species that were positive for UrsuERV and the corresponding ERV sequences are shown in red. Phylogenetic trees were generated using maximum likelihood analysis as implemented in RAxML [54]. The bootstrap consensus trees illustrated were inferred from 500 replicates with the percentage bootstrap given next to each branch. Evolutionary phylogeny comparison of host *versus* retrovirus illustrates lack of co-evolution and cross species transmission events.

Comparison of host and retroviral phylogenies can reveal discordances that provide evidence for cross species transmissions of retroviruses. If an ERV and its hosts have co-evolved, the species tree and retroviral tree should be largely concordant. Discordant trees then indicate retroviral introgression among lineages or independent infection of different lineages by the same or related retroviruses [53]. Comparison of the host and retroviral phylogenetic relationships indicated substantial cross species transmissions in all lineages. UrsusERVs identified in the bears do not exhibit a consistent host pathogen co evolutionary pattern consistent with multiple invasions of the bear lineage from an unknown reservoir (Figure 6).

## 3. Discussion

Next generation virome sequence analysis from the brains of two polar bears yielded sequences with similarity to PERV and MDEV. The identified short sequences were used to screen bear genome sequence data, eventually identifying full-length proviruses and solo LTRs belonging to a previously undescribed gammaretrovirus group of ERVs that is specific to the *Ursinae* subfamily of bears, thus designated UrsusERV. Prior to the advent of genomics, the effort required to fully characterize novel multi-copy retroviruses was substantial. The increasing availability of partially or fully annotated genomes from wildlife is facilitating the characterization of viral sequences by providing a reference for obtaining full length viral genomes based on fragmentary sequences identified by either standard or NGS based approaches. The age estimates for UrsusERV suggests the group, like some PERVs, enJSRVs and KoRV, is overall relatively young, which is also reflected by the relative intact nature of the provirus on scaffold 200 and potentially by the unusual low copy number of elements identified [55]. Genomic invasion occurred subsequent to the divergence of the bear lineages such that basal bear lineages that include giant pandas and spectacled bears are devoid of UrsusERV homologues. Dating of scaffold 162 was not possible due to the recombinant nature of the 3′ LTR, yet the non-recombined regions of the proviral LTRs, that are identical in sequence, indicate a very recent integration of that provirus as well. The LTRs of the provirus in scaffold 200 were identical in sequence, suggesting it is an evolutionarily very young ERV locus. In fact, the integration in scaffold 200 was polar bear specific. Although young, unlike KoRV and some enJSRVs, all UrsusERV loci are likely fully endogenized as the integration sites were fixed, among independent modern polar bear whole genome data examined (*n* = 6). Fewer were identified in draft genome sequence from an ancient polar bear. However, this more likely reflects the lower coverage of the ancient genome sequence data than actual absence.

Black bears carry sequences homologous to UrsusERV but none of the integration sites are orthologous. An UrsusERV nucleotide consensus sequence that was generated from the black bear whole genome data shared 93.5% identity to the polar bear UrsusERV consensus sequence. Phylogenetic analysis of the nucleotide sequences indicated that black bear consensus is basal to the UrsusERV clade (Figure S4). The basal position of the sequence and the lack of shared integration sites with brown and polar bears suggests that invasion of the black bear genome occurred independently from other ursids tested. However, we cannot rule out that ancient lineage sorting may explain the lack of orthologous integration among different bear species. All brown bears shared the proviral insertion in scaffold 162 consistent with the predicted date of divergence of polar bears and brown bears *ca*. one million years ago [50]. The provirus in scaffold 7, the oldest UrsusERV locus dated at approximately 2.1 million years, was present, based on draft sequence evidence, in the genomes of three out of four brown bears. The proviral integration on scaffold 1 was identified in two out of four brown bears. Sex chromosomes were excluded as the source of the polymorphism observed, as all brown bears examined were female. The polymorphic nature of these integrations in brown bears could indicate that the ancestor of brown bears was polymorphic for these insertions and different brown bear populations retained or lost the ERVs respectively. In contrast, according to our results, the provirus on polar bear scaffold 200 was absent from all brown bears suggesting invasion happened very recently and is exclusive to the polar bear lineage. The similarity to PERVs, the relative young age and the lack of congruence of viral homology and host specificity suggests that the exogenous UrsusERV equivalent was repeatedly in circulation over at least the last two million years from an unknown reservoir. The general lack of host and viral phylogenetic congruence observed suggests that UrsusERV related viruses have been independently infecting multiple mammalian lineages over the last 12 million years (Figure 6). Although generally basal in the phylogenetic trees, the killer whale endogenous retrovirus and UrsusERV show relatively strong affinity suggesting that a common reservoir for UrsusERV like viruses are present in the Arctic region. The very young nature of the provirus on scaffold 200 further suggests that exogenous forms could potentially still be in circulation.

The identification of unique LTR groups associated with different proviruses suggests there were invasions of at least two distinct UrsusERV variants. The phylogenetic positions of the scaffold 7 and 162 proviruses, which are basal in both the GAG and POL trees, suggest LTR-B containing viruses invaded the bear genome first. A subsequent invasion of LTR-A type variants represented by proviruses on scaffolds 1 and 200 followed, which have younger estimated integration ages and are more derived in the phylogenetic analysis. This may reflect a shift in viral strains circulating in the source species. The independence of the invasions is also suggested by the absence of orthologous integrations in the black bears for the polar bear scaffolds. Were the black bear loci orthologous with the polar bear loci, the age estimates would be vast underestimates given that the black bear lineage diverged from the polar bear and brown bear lineages at least 7 mya (Figure 5). If the invasions were independent as it seems, then the polar bear and brown bear UrsusERV younger age estimates are more consistent with divergence date estimates for the brown and polar bear clades. Confounding this analysis is the basal position of the provirus on scaffold 200 in the ENV phylogeny. The provirus on scaffold 1 could not be analyzed for ENV as the gene is deleted. This incongruity could represent recombination post integration or that the ERV is under selective pressure with the *env* gene having accumulated mutations [56]. An alternative explanation may be an assembly artifact as the scaffold 1 provirus harbors an assembly gap within the *pol* gene region. Even though independent invasions by LTR-A and LTR-B viruses is the most parsimonious explanation for the observed proviral distribution, the possibility of a single endogenization event, diversification and subsequent lineage sorting cannot be excluded as an explanation based on the current data.

UrsusERV occupied in all phylogenetic analyses a sister taxa position relative to a clade containing the PERVs, MDEV, McERV, GALVs and KoRVs. Only the ENV tree conflicted with this result placing McERV and MDEV in a basal position, which may reflect recombination events in those viruses. A consistent result across all analyses was the basal position of the killer whale endogenous retrovirus, which has an estimated endogenization date in the delphinoid genomes sometime in the past 12 million years [1].

With a young age estimate for PERVs, KoRVs and UrsusERV, and with GALV being only identified as an exogenous virus, the entire clade is relatively young in comparison to many other retroviral groups [47,57,58,59]. This suggests retroviruses with broad host range were disseminated across a wide variety of environments and species in the last 12 million years and, in the case of UrsusERV, it remains possible that exogenous forms related to the scaffold 200 provirus are still in circulation. As more next generation whole genome sequence data from wildlife becomes available and more endogenous retroviral genomes are characterized, the origins and distribution of gammaretroviruses such as UrsusERV will become clearer.

## 4. Materials and Methods

### 4.1. Samples

Polar bear tissue samples from Knut, a male polar bear, were provided by the Zoological Garden Berlin. Tissue samples from Jerka, a female polar bear, a spectacled bear (*Tremarctos ornatus)* and a black bear (*Ursus americanus*) were provided by the Zoological Garden Wuppertal, Tierpark Berlin, and Allwetterzoo Münster, respectively. All tissue samples were frozen at −80 °C until further processing.

### 4.2. Nucleic Acid Preparation and Next Generation Sequencing

DNA and RNA extraction from brain and liver bear tissue samples was performed with approximately 25 mg of tissue using a QIAamp DNA mini extraction kit and RNeasy Lipid kits following manufacturer instructions respectively. RNA sample preparation and next generation sequencing was performed as described in [23]. DNA and transcriptome data generated in [23] were used for data mining. Retroviral sequence data mined from the transcriptomes has been deposited in NCBI Sequence Read Archive (SRA) with accession number SRP065872.

### 4.3. UrsusERV Polymerase Chain Reaction in Multiple Bear Species

PCR products were amplified from genomic DNA from multiple bear tissues using the following primer pairs UrsusERV-F1 (5′-AGCCTACTTGGGATGACTGC-3′), UrsusERV-R1 (5′-GAGACCAGCCACTAGAGCCT-3′) and UrsusERV-F2 (5′-TAGAAGGGAGACAGATGAGGA-3′), UrsusERV-R2 (5′-TGAGAGTTATCCTGGGCTCA-3′). Polymerase chain reactions were performed in 50 μL reaction volumes containing 0.5 U of MyTaq HS polymerase mix (Bioline, Taunton, MA, USA), 400 nM of either paired primers, and 1 µL of DNA template. Thermocycling conditions were 95 °C denaturing for 3 min followed by 35 cycles of 95 °C for 15 s, 62 °C for 20 s, 72 °C for 15 s. Amplified products were visualized on 1.5% (*w*/*v*) agarose gels using Midori Green gel stain (Nippon Genetics, Dueren, Germany). Amplification products were sequenced using the forward and reverse primer used in the amplification (LGC Genomics, Berlin, Germany).

### 4.4. Mining of UrsusERV Sequences in the Polar Bear Genome

Viral database blast searches of the next generation sequence data of the two polar bear transcriptomes were performed as previously described [4]. Viral database screening results revealed reads with similarity to the *Mus dunni* endogenous retrovirus, Feline sarcoma virus, Murine leukemia virus, and to the Porcine endogenous retroviruses.

### 4.5. Generation of UrsusERV Provirus and LTR Consensus Sequences

Multiple alignments of the proviral amino acid sequence and LTR’s nucleotide sequence were generated using MAFFT v7.017 FFT-NS-i alignment strategy [52] as a plugin within the Geneious (Biomatters, Auckland, New Zealand) software. Resulting alignments were manually curated and a majority rule consensus was generated for each of the proviral genes as well as the LTRs. Retrotector [37] was employed to generate putative, reconstructed retroviral proteins, called puteins, for *Gag*, *Pro*, *Pol*, and *Env* genes based on the proviral consensus sequence. Conserved motifs in resulting consensus putein sequences were further verified using the NCBI Conserved Domain Database [38]. Proviral and LTR aligned sequences can be found in Supplementary fasta files 1 and S2. The UrsusERV consensus sequences can be found in RepBase under UrsusERV-A and B for the two LTR variants identified respectively and UrsusERV-I for the non-LTR sequences.

### 4.6. Recombination Inference Analysis

Recombination analysis was performed for multiple sequence alignments using three computational methods: Recombination Analysis Tool (RAT, Norwich, UK), Genetic Algorithm Recombination Detection (GARD), DualBrothers and RECCO [39,40,41,42,43]. GARD was used as implemented in the datamonkey online server [60] while DualBrothers (dual multiple change model) was used as a plugin in the Geneious software. RAT and GARD were used with default parameters, while DualBrothers was used with default model prior parameters and the following scanning window parameters: Window length = 300, Step size = 10. RECCO was implemented using the default parameters.

### 4.7. Sequence Read Archive and Bioinformatic Analysis

NCBI Sequence Read Archive data were used to determine whether the proviral sequences identified in the polar bear genome and the retroviral reads identified in the transcriptome were present in other bear species and whether the same proviral flanking regions were present in independent polar bear samples. Whole genome sequence data of *Ursus americanus* (accession number: SRR830685), *Ursus arctos* (SRR830213, SRR1693654, SRR1693624, SRR518712), *Ursus maritimus* (SRR827584-5, SRR827600, SRR947747, SRR942297), and ancient Poolepynten *Ursus maritimus* (SRR518649, SRR518651-7, SRR518704-5) were obtained. Short reads obtained were first converted to fastq format using sratoolkit version 2.3.5 [27]. Resulting fastq files were trimmed for adaptors and read quality using cutadapt version 1.5 and then were aligned to the draft polar bear genome using BWA 0.7.9a-r786 mem algorithm [49,61]. Resulting alignments were further curated using samtools 0.1.19 [62]. The identified proviral sequences from the draft polar bear genome were also used to search the Panda genome (*Ailuropoda melanoleuca*) by BLAT search at the UCSC Genome browser [48].

### 4.8. Phylogenetic Analysis

Multiple amino acid sequence alignments were generated using MAFFT version 7 and the iterative refinement method [52]. Further curation of the resulting alignments was performed manually. Phylogenetic analysis was performed using consensus amino acid sequences of the three major proteins (Gag, Pol and Env) of UrsusERV and related protein sequences obtained from GenBank: BaEV (accession numbers: BAP91048.1, BAA89659.1, YP_009109691.1), FeLV (NP_047255.1, NP_955577.1, NP_047256.1), F-MuLV (NP_040332.1, NP_040333.1, NP_040334.1), GaLV (NP_056789, NP_056790,NP_056791, AAA46809.1, AAA46810.1, AAA46811.1, AAC80263.1, AAC80264.1, AAC80265.1, AAC96086.1, AAC96085.1, AAC96084.1), KoRV (BAM67146.1, BAM67147.1), KoRV-Br2-1CEETG (AGO86849.1, AGO86848.1), McERV (KC460271.1, AGP25480.1, AGP25481.1), MDEV (AAC31804.1, AAC31805.1, AAC31806.1), M-CRV (NP_043518.1,NP_043519.1), MIRV (AFM52259.1, AFM52260.1), M-MuLV (NP_057933.1, NP_057935.1), OOEV (ACX69256.1, ACX69257.1), PERV-A (AEF12606.1, AAM29192.1,AEF12608.1), PERV-B (AAM29194.1, AAM29193.1), PERV-C (CAC39616.1, CAC39617.1, CAC39618.1), RaLV (Q83378, AAC78249.1, AAC78250.1), RD114 (YP_001497148.1, YP_001497149.1), REV (YP_223870.1, YP_223871.1, YP_223872.1), RfRV (AFA52558.1, AFA52559.1, AFA52560.1), RIRV (AFM52261.1, AFM52262.1), R-MuLV (NP_044737.1, NP_044738.1, NP_044739.1), SSV (AAC96087.1), WMSV (YP_001165469.2, YP_001165470.1, YP_003580185.1), DIAV(AGV92858.1, AGV92859.1, AGV92860.1), Echidna ERV (AGV92855.1, AGV92856.1), and Galidia ERV (AGV92852.1, AGV92853.1, AGV92854.1). Galidia ERV sequences were used as outgroup in the analysis. Protest version 3.4 was used to identify the best model for the phylogenetic analysis [63]. A Jones model with gamma distribution and invariable sites was determined as the optimal model for GAG and POL protein alignments, while for the ENV amino acid alignment the Rtrev model with invariable sites and gamma distributions was chosen. Bayesian inference analysis was performed as described in [64].

### 4.9. Age Estimation of UrsusERV Proviral Sequences

To estimate UrsusERV retroviral integration ages into the polar bear genome we used a molecular clock method. Proviral LTRs, upon integration into the host genome, are identical in sequence and they then acquire mutations independently from each other over time at the mutation rate of the host DNA [20]. Sequence divergence between the 5′ and 3′ LTRs of a provirus can thus serve as a molecular clock. Hypermutable CpGs sites were excluded from the age estimations. Sequence divergences were subsequently corrected using the Kimura-2-parameter (K2P) model [65]. The provirus formation dates were estimated using the formula T = D/2* 0.0015 per nucleotide and million years, where D is the corrected sequence divergence of the proviral 5′and 3′ LTRs and 0.0015/nt/myr is the reported mutation rate in polar bears [25].

### 4.10. Estimation of Divergence Times

Dating divergence events in the *Ursidae* family was performed using *cytb* and *irbp* gene sequences where available. The data sets were mined from the GenBank database with the following accession numbers: *Phoca vitulina* (AB188518.1; AB510422.1) *Ailuropoda melanoleuca* (EF212882.1; AY303836.1), *Tremarctos ornatus* (EF196665.1; AY303840.1), *Helarctos malayanus* (EF196664.1; AY303839.1), *Melursus ursinus* (EF196662.1; AY303838.1), *Ursus americanus* (AF303109.1; AY303837.1), *Ursus arctos* (KF184275.1; AY303842.1), *Ursus thibetanus* (EF196661.1; AY303841.1), *Ursus spelaeus* (AF264047.1), and *Ursus maritimus* (AF303111.1; AY303843.1). Dating of divergence times among the *Ursidae* clade was performed using a relaxed molecular clock approach along with the fossilized birth-death process in Beast v2.3.1 software [66,67,68] using priors from previous publications, and fossil calibration points [26,31,50]. Results were assessed using Tracer v1.6 and maximum likelihood trees were produced using Tree Annotator v2.3.1 [66]. Age estimation phylogenetic tree was visualized using R phylo package [69,70,71,72].

## Supplementary Materials

The following are available online at: http://www.mdpi.com/1999-4915/7/11/2927/s1.

## References

[1] Lamere S.A., st Leger J.A., Schrenzel M.D., Anthony S.J., Rideout B.A., Salomon D.R. (2009). Molecular characterization of a novel gammaretrovirus in killer whales (*Orcinus orca*). J. Virol..

[2] Wang L., Yin Q., He G., Rossiter S.J., Holmes E.C., Cui J. (2013). Ancient invasion of an extinct γ retrovirus in cetaceans. Virology.

[3] Gifford R.J. (2006). Evolution at the host-retrovirus interface. BioEssays.

[4] Katzourakis A., Rambaut A., Pybus O.G. (2005). The evolutionary dynamics of endogenous retroviruses. TIM.

[5] Mayer J., Tsangaras K., Heeger F., Avila-Arcos M., Stenglein M.D., Chen W., Sun W., Mazzoni C.J., Osterrieder N., Greenwood A.D. (2013). A novel endogenous betaretrovirus group characterized from polar bears (*Ursus maritimus*) and giant pandas (*Ailuropoda melanoleuca*). Virology.

[6] Niewiadomska A.M., Gifford R.J. (2013). The extraordinary evolutionary history of the reticuloendotheliosis viruses. PLoS Biol..

[7] Elleder D., Kim O., Padhi A., Bankert J.G., Simeonov I., Schuster S.C., Wittekindt N.E., Motameny S., Poss M. (2012). Polymorphic integrations of an endogenous γ retrovirus in the mule deer genome. J. Virol..

[8] Heidmann O., Vernochet C., Dupressoir A., Heidmann T. (2009). Identification of an endogenous retroviral envelope gene with fusogenic activity and placenta-specific expression in the rabbit: A new “syncytin” in a third order of mammals. Retrovirology.

[9] Mayer J., Meese E. (2005). Human endogenous retroviruses in the primate lineage and their influence on host genomes. Cytogenet. Genome Res..

[10] Tarlinton R.E., Meers J., Young P.R. (2006). Retroviral invasion of the koala genome. Nature.

[11] Arnaud F., Caporale M., Varela M., Biek R., Chessa B., Alberti A., Golder M., Mura M., Zhang Y.P., Yu L. (2007). A paradigm for virus-host coevolution: Sequential counter-adaptations between endogenous and exogenous retroviruses. PLoS Pathog..

[12] Avila-Arcos M.C., Ho S.Y., Ishida Y., Nikolaidis N., Tsangaras K., Honig K., Medina R., Rasmussen M., Fordyce S.L., Calvignac-Spencer S. (2013). One hundred twenty years of Koala retrovirus evolution determined from museum skins. Mol. Biol. Evol..

[13] Macfarlane C., Simmonds P. (2004). Allelic variation of HERV-K(HML-2) endogenous retroviral elements in human populations. J. Mol. Evol..

[14] Sistiaga-Poveda M., Jugo B.M. (2014). Evolutionary dynamics of endogenous jaagsiekte sheep retroviruses proliferation in the domestic sheep, mouflon and *Pyrenean chamois*. Heredity.

[15] Stoye J.P. (2006). Koala retrovirus: A genome invasion in real time. Genome Biol..

[16] Tsangaras K., Siracusa M.C., Nikolaidis N., Ishida Y., Cui P., Vielgrader H., Helgen K.M., Roca A.L., Greenwood A.D. (2014). Hybridization capture reveals evolution and conservation across the entire Koala retrovirus genome. PLoS ONE.

[17] Ishida Y., Zhao K., Greenwood A.D., Roca A.L. (2015). Proliferation of endogenous retroviruses in the early stages of a host germ line invasion. Mol. Biol. Evol..

[18] Oliveira N.M., Farrell K.B., Eiden M.V. (2006). *In vitro* characterization of a Koala retrovirus. J. Virol..

[19] Escalera-Zamudio M., Mendoza M.L., Heeger F., Loza-Rubio E., Rojas-Anaya E., Mendez-Ojeda M.L., Taboada B., Mazzoni C.J., Arias C.F., Greenwood A.D. (2015). A novel endogenous betaretrovirus in the common vampire bat (*Desmodus rotundus*) suggests multiple independent infection and cross-species transmission events. J. Virol..

[20] Cui J., Tachedjian G., Tachedjian M., Holmes E.C., Zhang S., Wang L.F. (2012). Identification of diverse groups of endogenous γ retroviruses in mega- and microbats. J. Gen. Virol..

[21] Li B., Zhang G., Willerslev E., Wang J., Wang J. (2011). Genomic data from the polar bear (*Ursus maritimus*). GigaScience.

[22] Li R., Fan W., Tian G., Zhu H., He L., Cai J., Huang Q., Cai Q., Li B., Bai Y. (2010). The sequence and *de novo* assembly of the giant panda genome. Nature.

[23] Szentiks C.A., Tsangaras K., Abendroth B., Scheuch M., Stenglein M.D., Wohlsein P., Heeger F., Hoveler R., Chen W., Sun W. (2014). Polar bear encephalitis: Establishment of a comprehensive next-generation pathogen analysis pipeline for captive and free-living wildlife. J. Comp. Pathol..

[24] Pages M., Calvignac S., Klein C., Paris M., Hughes S., Hanni C. (2008). Combined analysis of fourteen nuclear genes refines the *Ursidae* phylogeny. Mol. Phylogenet. Evol..

[25] Hailer F., Kutschera V.E., Hallstrom B.M., Klassert D., Fain S.R., Leonard J.A., Arnason U., Janke A. (2012). Nuclear genomic sequences reveal that polar bears are an old and distinct bear lineage. Science.

[26] Yu L., Li Q.W., Ryder O.A., Zhang Y.P. (2004). Phylogeny of the bears (*Ursidae*) based on nuclear and mitochondrial genes. Mol. Phylogenet. Evol..

[27] Leinonen R., Sugawara H., Shumway M., International Nucleotide Sequence Database Collaboration (2011). The sequence read archive. Nucleic Acids Res..

[28] Meyer M., Kircher M. (2010). Illumina sequencing library preparation for highly multiplexed target capture and sequencing. Cold Spring Harb. Protoc..

[29] Miller W., Schuster S.C., Welch A.J., Ratan A., Bedoya-Reina O.C., Zhao F., Kim H.L., Burhans R.C., Drautz D.I., Wittekindt N.E. (2012). Polar and brown bear genomes reveal ancient admixture and demographic footprints of past climate change. Proc. Natl. Acad. Sci. USA.

[30] Scobie L., Takeuchi Y. (2009). Porcine endogenous retrovirus and other viruses in xenotransplantation. Curr. Opin. Organ Transpl..

[31] Lindqvist C., Schuster S.C., Sun Y., Talbot S.L., Qi J., Ratan A., Tomsho L.P., Kasson L., Zeyl E., Aars J. (2010). Complete mitochondrial genome of a *Pleistocene jawbone* unveils the origin of polar bear. Proc. Natl. Acad. Sci. USA.

[32] Repeatmasker Open-3.0. http://www.repeatmasker.org.

[33] Sperber G., Lovgren A., Eriksson N.E., Benachenhou F., Blomberg J. (2009). Retrotector online, a rational tool for analysis of retroviral elements in small and medium size vertebrate genomic sequences. BMC Bioinform..

[34] Tempel S. (2012). Using and understanding repeatmasker. Methods Mol. Biol..

[35] Hanger J.J., Bromham L.D., McKee J.J., O’Brien T.M., Robinson W.F. (2000). The nucleotide sequence of Koala (*Phascolarctos cinereus*) retrovirus: A novel type C endogenous virus related to gibbon ape leukemia virus. J. Virol..

[36] Jurka J., Kapitonov V.V., Pavlicek A., Klonowski P., Kohany O., Walichiewicz J. (2005). Repbase update, a database of eukaryotic repetitive elements. Cytogenet. Genome Res..

[37] Sperber G.O., Airola T., Jern P., Blomberg J. (2007). Automated recognition of retroviral sequences in genomic data–retrotector. Nucleic Acids Res..

[38] Marchler-Bauer A., Lu S., Anderson J.B., Chitsaz F., Derbyshire M.K., DeWeese-Scott C., Fong J.H., Geer L.Y., Geer R.C., Gonzales N.R. (2011). CDD: A conserved domain database for the functional annotation of proteins. Nucleic Acids Res..

[39] Etherington G.J., Dicks J., Roberts I.N. (2005). Recombination analysis tool (RAT): A program for the high-throughput detection of recombination. Bioinformatics.

[40] Kosakovsky Pond S.L., Posada D., Gravenor M.B., Woelk C.H., Frost S.D. (2006). Gard: A genetic algorithm for recombination detection. Bioinformatics.

[41] Kosakovsky Pond S.L., Posada D., Gravenor M.B., Woelk C.H., Frost S.D.W. (2006). Automated phylogenetic detection of recombination using a genetic algorithm. Mol. Biol. Evol..

[42] Maydt J., Lengauer T. (2006). Recco: Recombination analysis using cost optimization. Bioinformatics.

[43] Minin V.N., Dorman K.S., Fang F., Suchard M.A. (2005). Dual multiple change-point model leads to more accurate recombination detection. Bioinformatics.

[44] Serrao E., Ballandras-Colas A., Cherepanov P., Maertens G.N., Engelman A.N. (2015). Key determinants of target DNA recognition by retroviral intasomes. Retrovirology.

[45] Belshaw R., Watson J., Katzourakis A., Howe A., Woolven-Allen J., Burt A., Tristem M. (2007). Rate of recombinational deletion among human endogenous retroviruses. J. Virol..

[46] Hughes J.F., Coffin J.M. (2005). Human endogenous retroviral elements as indicators of ectopic recombination events in the primate genome. Genetics.

[47] Stoye J.P. (2001). Endogenous retroviruses: Still active after all these years?. Curr. Biol..

[48] Karolchik D., Hinrichs A.S., Furey T.S., Roskin K.M., Sugnet C.W., Haussler D., Kent W.J. (2004). The UCSC table browser data retrieval tool. Nucleic Acids Res..

[49] Li H., Durbin R. (2009). Fast and accurate short read alignment with burrows-wheeler transform. Bioinformatics.

[50] Krause J., Unger T., Nocon A., Malaspinas A.S., Kolokotronis S.O., Stiller M., Soibelzon L., Spriggs H., Dear P.H., Briggs A.W. (2008). Mitochondrial genomes reveal an explosive radiation of extinct and extant bears near the miocene-pliocene boundary. BMC Evol. Biol..

[51] Cahill J.A., Stirling I., Kistler L., Salamzade R., Ersmark E., Fulton T.L., Stiller M., Green R.E., Shapiro B. (2015). Genomic evidence of geographically widespread effect of gene flow from polar bears into brown bears. Mol. Ecol..

[52] Katoh K., Misawa K., Kuma K., Miyata T. (2002). Mafft: A novel method for rapid multiple sequence alignment based on fast fourier transform. Nucleic Acids Res..

[53] Hayward A., Grabherr M., Jern P. (2013). Broad-scale phylogenomics provides insights into retrovirus-host evolution. Proc. Natl. Acad. Sci. USA.

[54] Stamatakis A. (2006). RAxML-VI-HPC: Maximum likelihood-based phylogenetic analyses with thousands of taxa and mixed models. Bioinformatics.

[55] Tonjes R.R., Niebert M. (2003). Relative age of proviral porcine endogenous retrovirus sequences in *Sus scrofa* based on the molecular clock hypothesis. J. Virol..

[56] Magiorkinis G., Gifford R.J., Katzourakis A., de Ranter J., Belshaw R. (2012). Env-less endogenous retroviruses are genomic superspreaders. Proc. Natl. Acad. Sci. USA.

[57] Flockerzi A., Burkhardt S., Schempp W., Meese E., Mayer J. (2005). Human endogenous retrovirus HERV-K14 families: Status, variants, evolution, and mobilization of other cellular sequences. J. Virol..

[58] Gifford R., Tristem M. (2003). The evolution, distribution and diversity of endogenous retroviruses. Virus Genes.

[59] Schrago C.G., Russo C.A. (2003). Timing the origin of new world monkeys. Mol. Biol. Evol..

[60] Delport W., Poon A.F., Frost S.D., Kosakovsky Pond S.L. (2010). Datamonkey 2010: A suite of phylogenetic analysis tools for evolutionary biology. Bioinformatics.

[61] Marcel M. (2011). Cutadapt removes adapter sequences from high-throughput sequencing reads. EMBnet. J..

[62] Li H., Handsaker B., Wysoker A., Fennell T., Ruan J., Homer N., Marth G., Abecasis G., Durbin R., 1000 Genome Project Data Processing Subgroup (2009). The sequence alignment/map format and samtools. Bioinformatics.

[63] Darriba D., Taboada G.L., Doallo R., Posada D. (2011). Prottest 3: Fast selection of best-fit models of protein evolution. Bioinformatics.

[64] Huelsenbeck J.P., Ronquist F. (2001). Mrbayes: Bayesian inference of phylogenetic trees. Bioinformatics.

[65] Kimura M. (1980). A simple method for estimating evolutionary rates of base substitutions through comparative studies of nucleotide sequences. J. Mol. Evol..

[66] Bouckaert R., Heled J., Kuhnert D., Vaughan T., Wu C.H., Xie D., Suchard M.A., Rambaut A., Drummond A.J. (2014). Beast 2: A software platform for bayesian evolutionary analysis. PLoS Comput. Biol..

[67] Heath T.A., Huelsenbeck J.P., Stadler T. (2014). The fossilized birth-death process for coherent calibration of divergence-time estimates. Proc. Natl. Acad. Sci. USA.

[68] Drummond A.J., Ho S.Y., Phillips M.J., Rambaut A. (2006). Relaxed phylogenetics and dating with confidence. PLoS Biol..

[69] R: A Language and Environment for Statistical Computing. http://www.R-project.org2013.

[70] Revell L.J. (2012). Phytools: An r package for phylogenetic comparative biology (and other things). Methods Ecol. Evol..

[71] Heibl C. (2008). Phyloch: R language tree plotting tools and interfaces to diverse phylogenetic software packages.

[72] Bell M.A., Lloyd G.T. (2015). Strap: An R package for plotting phylogenies against stratigraphy and assessing their stratigraphic congruence. Palaeo.

