# Supplementary material for: An Evolutionarily Young Polar Bear (Ursus maritimus) Endogenous Retrovirus Identified from Next Generation Sequence Data
Source: Viruses. 2015 Nov 24;7(11):6089–107. doi: 10.3390/v7112927 (PMC4664997; doi:10.3390/v7112927)
Supplement: Supplementary file 1 [file viruses-07-02927-s001.zip › viruses-100334-Supplementary File/viruses-100334-Supplementary Figures and Table S1.docx]

An Evolutionarily Young Polar Bear (*Ursus maritimus*) Endogenous Retrovirus Identified from Next Generation Sequence Data

Kyriakos Tsangaras, Jens Mayer, David E. Alquezar-Planas and Alex D. Greenwood

**Figure S1: Recombination analysis of LTR sequences.(A)** DataMonkey GARD breakpoint analysis using the LTR alignment as input indicates a breakpoint in the 209 bp of the LTR alignment [1]. (**B)** GARD phylogenetic analysis using the first 209 bp of the analysis places the Scaffold 162 LTR A/B sequence the LTR – B subgroup, while phylogenetic analysis of the sequence after the identified breakpoint places Scaffold 162 LTR A/B sequence in the LTR-A subgroup. Indicating that the identified breakpoint is located in Scaffold 162 LTR A/B sequence [1]. **C)** DualBrother recombination analysis identifies a breakpoint in the 209 bp of the alignment. Sequence variation indicates that the breakpoint is in Scaffold 162 LTR A/B sequence [2].

**Figure S2: Amplified Sequences obtained by PCR and Sanger sequencing from multiple bear species.** PCR amplification of GAG gene was performed on DNA extractions from *Ailuropoda melanoleuca, Tremactos ornatus, Ursus americanus, Ursus arctos syriacus*, and *Ursus maritimus*. Amplified product were obtained for all species except the giant panda and the spectacled bear. UrsusERV homologues sequences are demonstrated for *Ursus americanus*, and *Ursus arctos syriacus* bear species.


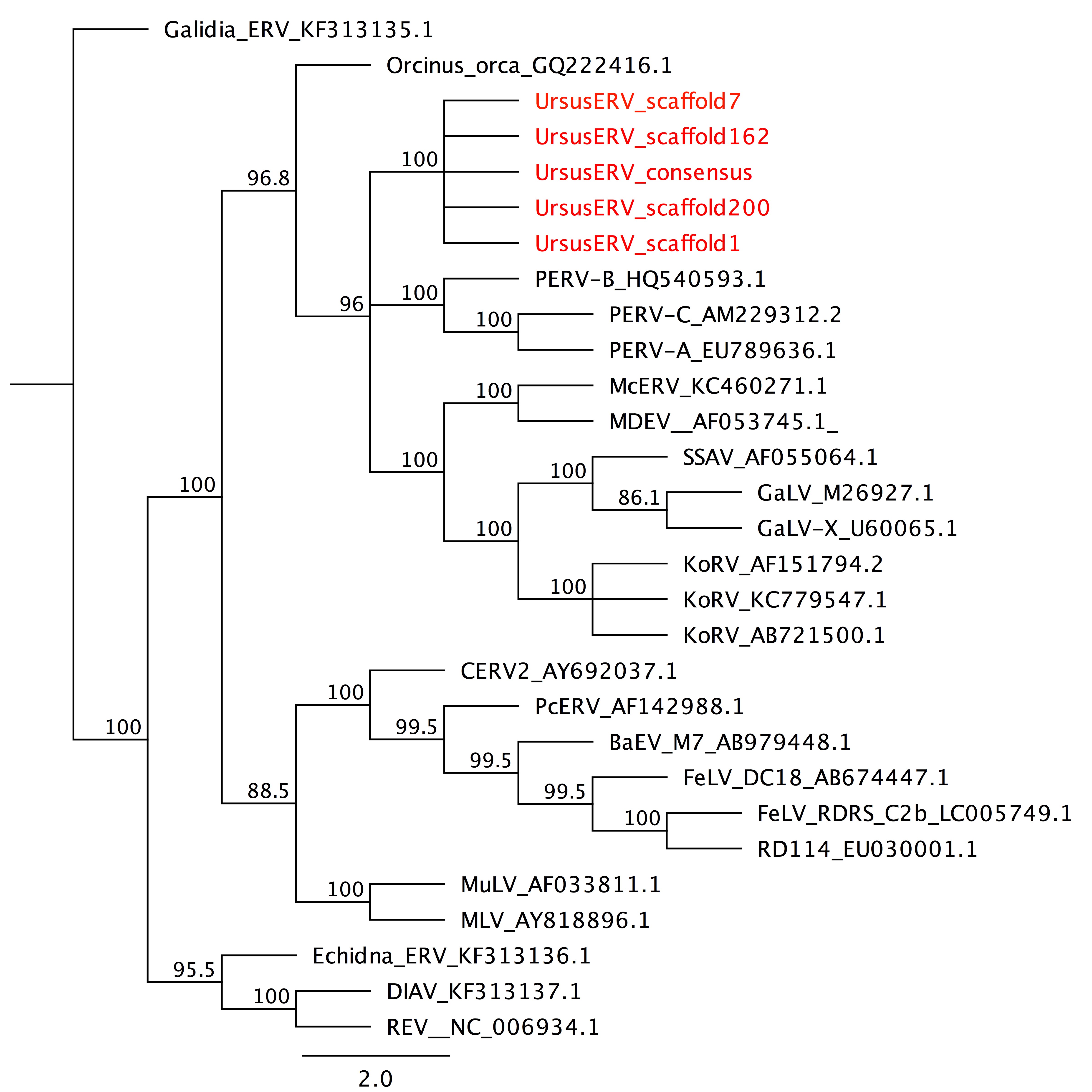


**Figure S3.** Maximum likelihood analysis of UrsusERV, PERVs, GaLVs, KoRVs and other γ retroviral nucleotide sequence. Multiple alignment of nucleotide sequences was performed using MAFFT default parameters [3,4]. The resulting alignment was inspected and curated manually. A region that corresponded to gag, and pol genes was used for phylogenetic analysis by maximum likelihood as implemented in RAxML [5]. Shown here is a bootstrap consensus tree inferred from 500 replicates with the percentage bootstrap support given next to each branch. UrsusERV sequences are highlighted in red.


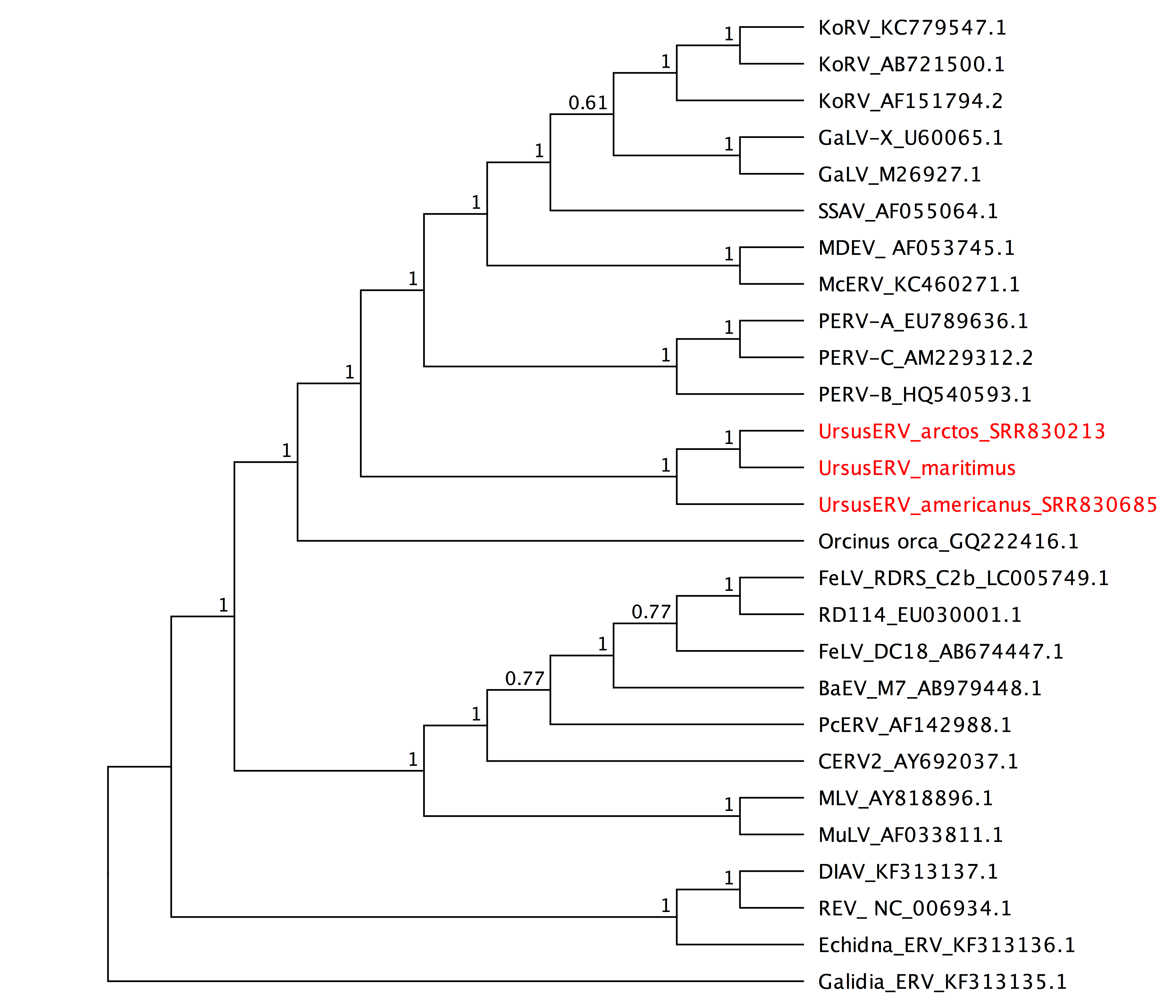


**Figure S4.** Bayesian analysis of UrsusERV nucleotide consensus obtained from polar, brown, and black bears and other gammaretroviral nucleotide sequences. Multiple alignment of nucleotide sequences was performed using MAFFT default parameters [3,4]. The resulting alignment was inspected and curated manually. A region that corresponded to gag, pol, and env genes was used for Bayesian phylogenetic analysis [6]. Shown here is a MCMC consensus tree inferred from 1000000 replicates with the posterior probability given next to each branch. UrsusERV consensus sequences are highlighted in red.

**Table S1.** UrsusERV provirus harboring regions identified within *Ursus maritimus* draft genome scaffold sequences using UrsusERV consensus as query sequence.

| **Hit name** | **Hit start** | **Hit stop** | **Query start** | **Query stop** | **Query coverage** | **% Pairwise Identity** | **E value** |
| --- | --- | --- | --- | --- | --- | --- | --- |
| scaffold1 | 66677104 | 66680923 | 1 | 3811 | 61.85% | 98.70% | 0 |
| scaffold7 | 30626432 | 30620270 | 1 | 6162 | 100.00% | 95.90% | 0 |
| scaffold162 | 4550 | 6899 | 3813 | 6162 | 38.14% | 99.80% | 0 |
| scaffold162 | 8384 | 10330 | 1 | 1947 | 31.60% | 99.30% | 0 |
| scaffold200 | 145718 | 151388 | 17 | 5693 | 92.13% | 97.70% | 0 |
| scaffold200 | 152216 | 152695 | 5682 | 6162 | 7.81% | 92.90% | 0 |

**Table S2**: Repeatmasker result of UrsusERV presumed harboring loci. UrsusERV presumed harboring loci including ~15,000 bp both upstream and downstream where screened with repeatmasker [7,8]. UrsusERV LTRs sequences are illustrated in green, while UrsusERV proviral main body is indicated with red (See Table S2 in Separate file attached).

**References**

1. Kosakovsky Pond, S.L.; Posada, D.; Gravenor, M.B.; Woelk, C.H.; Frost, S.D. Gard: A genetic algorithm for recombination detection. *Bioinformatics* **2006**, *22*, 3096-3098.

2. Minin, V.N.; Dorman, K.S.; Fang, F.; Suchard, M.A. Dual multiple change-point model leads to more accurate recombination detection. *Bioinformatics* **2005**, *21*, 3034-3042.

3. Katoh, K.; Kuma, K.; Toh, H.; Miyata, T. Mafft version 5: Improvement in accuracy of multiple sequence alignment. *Nucleic Acids Res* **2005**, *33*, 511-518.

4. Katoh, K.; Misawa, K.; Kuma, K.; Miyata, T. Mafft: A novel method for rapid multiple sequence alignment based on fast fourier transform. *Nucleic Acids Res* **2002**, *30*, 3059-3066.

5. Stamatakis, A. Raxml-vi-hpc: Maximum likelihood-based phylogenetic analyses with thousands of taxa and mixed models. *Bioinformatics* **2006**, *22*, 2688-2690.

6. Huelsenbeck, J.P.; Ronquist, F. Mrbayes: Bayesian inference of phylogenetic trees. Bioinformatics 2001, 17, 754-755.

7. open-3.0. URL:http://www.repeatmasker.org (accessed on 1 September 2015).

8. Tempel, S. Using and understanding repeatmasker. *Methods Mol Biol* **2012**,*859*, 29-51.
