# Supplementary material for: An Evolutionarily Young Polar Bear (Ursus maritimus) Endogenous Retrovirus Identified from Next Generation Sequence Data
Source: Viruses. 2015 Nov 24;7(11):6089–107. doi: 10.3390/v7112927 (PMC4664997; doi:10.3390/v7112927)
Supplement: Supplementary file 1 [file viruses-07-02927-s001.zip › viruses-100334-Supplementary File/viruses-100334-Table S2-Repeatmasker result of UrsusERV presumed harboring loci..pdf]

SW score	perc div.	perc del.	perc ins.	query sequence	position in query begin	end	(left)		matching repeat	repeat class/family	position in repeat begin	end	(left)	ID	
375	26.8	2.6	6.2	scaffold143_3,240,578-3,261,249	7	157	(20515)	C	LTR11B_AMe	LTR/ERV1	(331)	146	1	1	
373	33.3	0.0	0.0	scaffold143_3,240,578-3,261,249	158	298	(20374)	C	LTR77-int_TS	LTR/ERV1	(0)	5551	5411	2	
826	15.7	11.6	5.5	scaffold143_3,240,578-3,261,249	2025	2213	(18459)	C	SINEC1_AMe	SINE/tRNA	(0)	201	2	3	
329	23.3	15.8	0.0	scaffold143_3,240,578-3,261,249	2652	2771	(17901)	C	L1M5	LINE/L1	(1354)	4792	4654	4	
13	16.9	0.0	5.7	scaffold143_3,240,578-3,261,249	2915	2951	(17721)	+	(TTTC)n	Simple_repeat	1	35	(0)	5	
413	15.2	1.3	0.0	scaffold143_3,240,578-3,261,249	3229	3307	(17365)	C	MLT1E2	LTR/ERVL-MaLR	(0)	627	548	6	*
845	21.0	25.6	6.5	scaffold143_3,240,578-3,261,249	3306	3671	(17001)	C	MLT1E2	LTR/ERVL-MaLR	(191)	436	1	6	
2778	11.5	1.9	2.7	scaffold143_3,240,578-3,261,249	3711	4163	(16509)	+	CarLTR7	LTR/ERV1	1	433	(50)	7	
1491	4.7	3.1	0.5	scaffold143_3,240,578-3,261,249	4164	4357	(16315)	C	SINEC1_AMe	SINE/tRNA	(0)	201	3	8	
1032	7.7	0.1	20.2	scaffold143_3,240,578-3,261,249	4358	4441	(16231)	+	CarLTR7	LTR/ERV1	415	483	(0)	7	
1395	22.1	5.1	4.9	scaffold143_3,240,578-3,261,249	4444	4498	(16174)	+	Charlie4a	DNA/hAT-Charlie	60	116	(392)	9	
2286	15.5	6.3	4.7	scaffold143_3,240,578-3,261,249	4499	4958	(15714)	C	MER101B_CF	LTR/ERV1	(0)	467	1	10	
1395	22.1	5.1	4.9	scaffold143_3,240,578-3,261,249	4959	5350	(15322)	+	Charlie4a	DNA/hAT-Charlie	117	508	(0)	9	
473	27.5	11.2	0.0	scaffold143_3,240,578-3,261,249	5465	5624	(15048)	C	MLT1J	LTR/ERVL-MaLR	(315)	197	20	11	
315	14.7	7.9	1.2	scaffold143_3,240,578-3,261,249	5745	5820	(14852)	C	SINEC_c2	SINE/tRNA	(0)	212	132	12	
480	21.6	11.7	0.0	scaffold143_3,240,578-3,261,249	5822	5932	(14740)	C	SINEC2_AMe	SINE/tRNA	(9)	124	1	13	
646	7.8	50.0	1.0	scaffold143_3,240,578-3,261,249	6217	6346	(14326)	+	SINEC_b2	SINE/tRNA	1	193	(1)	14	
512	26.5	5.3	1.9	scaffold143_3,240,578-3,261,249	6448	6597	(14075)	C	LTR86C	LTR/ERVL	(2)	619	465	15	
354	29.9	4.7	0.0	scaffold143_3,240,578-3,261,249	7338	7444	(13228)	C	MIR	SINE/MIR	(104)	158	47	16	
379	22.4	3.9	14.1	scaffold143_3,240,578-3,261,249	7512	7667	(13005)	+	MER92D	LTR/ERV1	179	320	(0)	17	
616	28.4	1.8	0.0	scaffold143_3,240,578-3,261,249	7708	7876	(12796)	+	MER5A	DNA/hAT-Charlie	18	189	(0)	18	
285	32.1	7.1	0.0	scaffold143_3,240,578-3,261,249	9845	9956	(10716)	C	PlatSat2A	Satellite	(671)	362	243	19	
465	22.1	3.7	2.0	scaffold143_3,240,578-3,261,249	10044	10220	(10452)	C	LTRIS4	LTR/ERV1	(45)	462	205	20	
1463	7.8	3.1	0.0	scaffold143_3,240,578-3,261,249	10942	11134	(9538)	C	SINEC1_AMe	SINE/tRNA	(2)	199	1	21	
560	22.1	7.6	0.7	scaffold143_3,240,578-3,261,249	11329	11460	(9212)	+	SINEC1B_AMe	SINE/tRNA	1	141	(87)	22	
370	28.7	8.4	4.5	scaffold143_3,240,578-3,261,249	12118	12332	(8340)	C	MLT1K	LTR/ERVL-MaLR	(63)	532	310	23	
1250	19.7	4.6	5.8	scaffold143_3,240,578-3,261,249	12440	12612	(8060)	C	L1MB8	LINE/L1	(0)	6178	6015	24	
2449	14.9	6.8	0.9	scaffold143_3,240,578-3,261,249	12613	13053	(7619)	C	MER101B_CF	LTR/ERV1	(0)	467	1	25	
1250	19.7	4.6	5.8	scaffold143_3,240,578-3,261,249	13054	13245	(7427)	C	L1MB8	LINE/L1	(164)	6014	5817	24	
259	32.2	9.1	2.9	scaffold143_3,240,578-3,261,249	13246	13475	(7197)	C	Sat-1_TSy	Satellite	(316)	2920	2677	26	
268	18.1	5.0	11.7	scaffold143_3,240,578-3,261,249	13477	13576	(7096)	C	L1M3	LINE/L1	(2430)	5565	5472	27	*
1832	24.4	7.3	5.3	scaffold143_3,240,578-3,261,249	13554	14007	(6665)	C	L1M4	LINE/L1	(1485)	4661	4486	28	
1496	7.6	1.5	0.0	scaffold143_3,240,578-3,261,249	14008	14205	(6467)	C	SINEC1_AMe	SINE/tRNA	(0)	201	1	29	
2408	22.1	3.4	4.7	scaffold143_3,240,578-3,261,249	14206	14233	(6439)	C	L1M4	LINE/L1	(1668)	4478	4448	30	
605	15.7	3.7	0.0	scaffold143_3,240,578-3,261,249	14234	14341	(6331)	C	SINEC_old	SINE/tRNA	(0)	112	1	31	
2408	21.6	4.2	6.9	scaffold143_3,240,578-3,261,249	14342	14967	(5705)	C	L1M4	LINE/L1	(1841)	4504	3848	28	
1487	6.2	3.1	0.0	scaffold143_3,240,578-3,261,249	14968	15162	(5510)	C	SINEC1_AMe	SINE/tRNA	(0)	201	1	32	
1965	20.0	5.1	9.5	scaffold143_3,240,578-3,261,249	15163	15241	(5431)	C	L1M4	LINE/L1	(2299)	3847	3779	28	
1496	7.5	1.0	1.0	scaffold143_3,240,578-3,261,249	15242	15442	(5230)	C	SINEC1_AMe	SINE/tRNA	(0)	201	1	33	
1965	20.0	5.1	9.5	scaffold143_3,240,578-3,261,249	15443	15601	(5071)	C	L1M4	LINE/L1	(2368)	3778	3626	28	
1617	6.5	1.0	0.0	scaffold143_3,240,578-3,261,249	15602	15800	(4872)	+	SINEC1_AMe	SINE/tRNA	1	201	(0)	34	
1965	20.0	5.1	9.5	scaffold143_3,240,578-3,261,249	15801	16016	(4656)	C	L1M4	LINE/L1	(2521)	3625	3411	28	
4514	20.5	2.4	2.3	scaffold143_3,240,578-3,261,249	16017	16928	(3744)	C	LTR21B2_EC	LTR/ERVL	(0)	913	1	35	
963	15.7	10.8	0.0	scaffold143_3,240,578-3,261,249	16929	17151	(3521)	C	L1M4	LINE/L1	(2740)	3406	3160	28	
307	18.3	4.0	4.0	scaffold143_3,240,578-3,261,249	17315	17388	(3284)	C	Charlie1b	DNA/hAT-Charlie	(19)	504	431	36	
293	21.7	5.7	5.7	scaffold143_3,240,578-3,261,249	18714	18801	(1871)	+	MER70-int	LTR/ERVL	4578	4665	(358)	37	
479	29.2	4.1	0.7	scaffold143_3,240,578-3,261,249	19106	19250	(1422)	+	LTR75B	LTR/ERVL	1	150	(458)	38	
267	22.2	7.5	5.9	scaffold143_3,240,578-3,261,249	19313	19379	(1293)	+	LTR75B	LTR/ERVL	262	329	(233)	38	
218	32.1	3.7	0.0	scaffold143_3,240,578-3,261,249	19572	19652	(1020)	+	MIRb	SINE/MIR	177	260	(8)	39	
748	15.2	17.6	3.0	scaffold143_3,240,578-3,261,249	19879	20054	(618)	C	SINEC1_AMe	SINE/tRNA	(0)	201	1	40	
18	17.9	0.0	0.0	scaffold143_3,240,578-3,261,249	20206	20244	(428)	+	(T)n	Simple_repeat	1	39	(0)	41	
188	16.7	18.8	0.0	scaffold143_3,240,578-3,261,249	20439	20486	(186)	C	L2a	LINE/L2	(1)	3425	3369	42	
258	28.0	10.9	0.9	scaffold143_3,240,578-3,261,249	20503	20603	(69)	+	MER94	DNA/hAT-Blackjack	23	133	(1)	43	
1980	17.5	5.6	0.0	scaffold155_1,113,688-1,134,337	2	361	(20289)	C	CarLTR2	LTR/ERV1	(198)	380	1	44	
427	16.7	0.0	4.2	scaffold155_1,113,688-1,134,337	362	436	(20214)	C	MER68-int	LTR/ERVL	(2656)	334	263	45	
515	25.8	2.7	2.1	scaffold155_1,113,688-1,134,337	545	730	(19920)	C	MER68-int	LTR/ERVL	(2795)	195	9	45	
338	8.9	0.0	2.2	scaffold155_1,113,688-1,134,337	777	822	(19828)	+	LTR31_AMe	LTR/ERV1	1	45	(479)	46	
666	19.3	11.4	4.1	scaffold155_1,113,688-1,134,337	823	1006	(19644)	+	SINEC1_AMe	SINE/tRNA	1	197	(4)	47	
948	14.2	8.3	4.5	scaffold155_1,113,688-1,134,337	1035	1226	(19424)	C	SINEC1_AMe	SINE/tRNA	(2)	199	1	48	
1608	6.0	0.0	1.0	scaffold155_1,113,688-1,134,337	1311	1513	(19137)	+	SINEC1_AMe	SINE/tRNA	1	201	(0)	49	
781	18.9	3.4	7.1	scaffold155_1,113,688-1,134,337	1781	1985	(18665)	+	SINEC1_AMe	SINE/tRNA	2	199	(2)	50	
918	12.9	12.5	3.0	scaffold155_1,113,688-1,134,337	1989	2172	(18478)	+	SINEC1_AMe	SINE/tRNA	1	201	(0)	51	
833	13.1	7.7	5.6	scaffold155_1,113,688-1,134,337	2190	2383	(18267)	C	SINEC1_AMe	SINE/tRNA	(3)	198	1	52	
2685	9.6	0.7	3.2	scaffold155_1,113,688-1,134,337	2390	2799	(17851)	C	L1_Carn3	LINE/L1	(8)	6429	6030	53	
1116	32.5	2.9	1.8	scaffold155_1,113,688-1,134,337	2992	3483	(17167)	+	L1MEd	LINE/L1	416	912	(6209)	54	
572	21.4	4.3	8.5	scaffold155_1,113,688-1,134,337	3484	3692	(16958)	+	SINEC1_AMe	SINE/tRNA	1	201	(0)	55	
1573	7.0	0.0	0.0	scaffold155_1,113,688-1,134,337	4153	4351	(16299)	+	SINEC1_AMe	SINE/tRNA	1	199	(2)	56	
877	11.9	20.6	0.0	scaffold155_1,113,688-1,134,337	4724	4883	(15767)	C	SINEC_c1	SINE/tRNA	(16)	193	1	57	
265	21.9	1.0	3.1	scaffold155_1,113,688-1,134,337	4884	4973	(15677)	+	L1MEd	LINE/L1	882	970	(6151)	54	
518	16.5	7.7	0.0	scaffold155_1,113,688-1,134,337	4974	5064	(15586)	+	SINEC_old	SINE/tRNA	1	98	(14)	58	
1365	13.1	0.0	0.0	scaffold155_1,113,688-1,134,337	5074	5271	(15379)	+	SINEC1_AMe	SINE/tRNA	1	198	(3)	59	
1049	12.5	8.9	3.0	scaffold155_1,113,688-1,134,337	5281	5470	(15180)	+	SINEC1_AMe	SINE/tRNA	1	201	(0)	60	
1037	19.9	4.4	1.1	scaffold155_1,113,688-1,134,337	5495	5811	(14839)	+	L1MEd	LINE/L1	947	1297	(5824)	54	
1851	21.8	1.8	9.0	scaffold155_1,113,688-1,134,337	5812	5880	(14770)	C	MLT1C	LTR/ERVL-MaLR	(0)	467	409	61	
765	15.9	4.9	7.5	scaffold155_1,113,688-1,134,337	5881	6084	(14566)	C	SINEC1_AMe	SINE/tRNA	(2)	199	1	62	
1851	21.8	1.8	9.0	scaffold155_1,113,688-1,134,337	6085	6391	(14259)	C	MLT1C	LTR/ERVL-MaLR	(59)	408	107	61	
4214	7.7	0.8	3.1	scaffold155_1,113,688-1,134,337	6392	6981	(13669)	C	CarLTR1B1	LTR/ERV1	(0)	579	3	63	
1851	21.8	1.8	9.0	scaffold155_1,113,688-1,134,337	6982	7103	(13547)	C	MLT1C	LTR/ERVL-MaLR	(361)	106	1	61	
486	21.7	5.8	9.3	scaffold155_1,113,688-1,134,337	7105	7172	(13478)	+	L1MEd	LINE/L1	1297	1350	(5771)	54	
1563	7.5	0.0	0.0	scaffold155_1,113,688-1,134,337	7173	7373	(13277)	+	SINEC1_AMe	SINE/tRNA	1	201	(0)	64	
486	21.7	5.8	9.3	scaffold155_1,113,688-1,134,337	7374	7527	(13123)	+	L1MEd	LINE/L1	1316	1477	(5609)	65	
523	20.2	1.8	0.9	scaffold155_1,113,688-1,134,337	7528	7637	(13013)	+	SINEC_old	SINE/tRNA	1	111	(1)	66	
810	19.1	8.2	3.4	scaffold155_1,113,688-1,134,337	7638	7832	(12818)	+	SINEC_c1	SINE/tRNA	6	209	(0)	67	
248	5.0	3.2	4.8	scaffold155_1,113,688-1,134,337	7782	7834	(12816)	+	B2L_S	SINE/B4	164	216	(38)	68	*
961	14.9	6.7	2.5	scaffold155_1,113,688-1,134,337	7835	8027	(12623)	+	SINEC1_AMe	SINE/tRNA	1	201	(0)	69	
444	20.6	14.1	2.4	scaffold155_1,113,688-1,134,337	8037	8185	(12465)	+	L1MEd	LINE/L1	1450	1615	(617)	65	
289	27.4	0.0	0.0	scaffold155_1,113,688-1,134,337	8222	8294	(12356)	+	MARNA	DNA/TcMar-Mariner	316	388	(198)	70	
1531	4.7	4.2	0.0	scaffold155_1,113,688-1,134,337	8337	8529	(12121)	C	SINEC1_AMe	SINE/tRNA	(0)	201	1	71	
302	18.8	12.5	0.0	scaffold155_1,113,688-1,134,337	8567	8630	(12020)	+	tRNA-Lys-AAG	tRNA	2	73	(3)	72	
824	10.2	18.0	9.5	scaffold155_1,113,688-1,134,337	8738	8943	(11707)	+	SINEC1B_AMe	SINE/tRNA	1	222	(6)	73	
441	25.8	3.9	5.0	scaffold155_1,113,688-1,134,337	8949	8991	(11659)	+	L1MEd	LINE/L1	4990	5025	(1121)	54	
959	9.3	18.6	0.0	scaffold155_1,113,688-1,134,337	8992	9152	(11498)	+	SINEC_b1	SINE/tRNA	2	192	(2)	74	
441	25.8	3.9	5.0	scaffold155_1,113,688-1,134,337	9153	9312	(11338)	+	L1MEd	LINE/L1	5026	5190	(956)	54	*
825	6.8	0.0	15.7	scaffold155_1,113,688-1,134,337	9296	9374	(11276)	+	L1_Canid_	LINE/L1	5254	5316	(1135)	75	
1003	11.3	0.0	0.8	scaffold155_1,113,688-1,134,337	9375	9508	(11142)	C	SINEC2_AMe	SINE/tRNA	(0)	133	1	76	
825	6.8	0.0	15.7	scaffold155_1,113,688-1,134,337	9509	9568	(11082)	+	L1_Canid_	LINE/L1	5317	5374	(1077)	75	
287	31.4	4.2	8.4	scaffold155_1,113,688-1,134,337	9569	9632	(11018)	+	Sat-1_TSy	Satellite	2253	2313	(923)	77	
1135	10.9	17.7	0.0	scaffold155_1,113,688-1,134,337	9633	9824	(10826)	+	SINEC1B_AMe	SINE/tRNA	1	226	(2)	78	
287	31.4	4.2	8.4	scaffold155_1,113,688-1,134,337	9825	9995	(10655)	+	Sat-1_TSy	Satellite	2314	2479	(757)	77	
446	23.2	4.3	5.4	scaffold155_1,113,688-1,134,337	10012	10220	(10430)	C	LTRIS4A	LTR/ERV1	(42)	432	205	79	
449	32.5	2.8	3.4	scaffold155_1,113,688-1,134,337	10677	11065	(9585)	+	Sat-1_TSy	Satellite	2516	2902	(334)	77	
418	16.7	1.4	0.0	scaffold155_1,113,688-1,134,337	11128	11199	(9451)	C	SINEC_old	SINE/tRNA	(11)	101	29	80	
294	17.0	0.0	0.0	scaffold155_1,113,688-1,134,337	11210	11256	(9394)	+	LTR51_CF	LTR/ERV1	31	77	(548)	81	
559	23.5	6.4	6.0	scaffold155_1,113,688-1,134,337	11264	11512	(9138)	+	LTR51_CF	LTR/ERV1	373	622	(3)	81	
670	31.8	1.4	0.7	scaffold155_1,113,688-1,134,337	11911	12201	(8449)	+	PABL_B-int	LTR/ERV1	464	756	(5040)	82	
493	29.6	6.2	4.9	scaffold155_1,113,688-1,134,337	11999	12240	(8410)	+	HERV17-int	LTR/ERV1	2027	2271	(6355)	83	*
838	33.1	3.1	3.0	scaffold155_1,113,688-1,134,337	12414	13064	(7586)	+	PABL_B-int	LTR/ERV1	1098	1887	(5471)	82	
256	23.6	0.0	0.0	scaffold155_1,113,688-1,134,337	13066	13120	(7530)	+	PABL_B-int	LTR/ERV1	1607	1661	(5763)	82	
304	13.6	1.2	9.3	scaffold155_1,113,688-1,134,337	13334	13414	(7236)	+	ERV1-2_OC-I	LTR/ERV1	1901	1965	(3234)	84	
338	10.8	8.3	0.0	scaffold155_1,113,688-1,134,337	13655	13722	(6928)	+	SINEC_b2	SINE/tRNA	1	86	(108)	85	*
585	23.2	9.8	2.2	scaffold155_1,113,688-1,134,337	13711	13931	(6719)	+	Mer34_Ttr	LTR/ERV1	317	538	(5)	86	
1651	22.3	9.3	5.1	scaffold155_1,113,688-1,134,337	13935	14745	(5905)	+	MER34-int	LTR/ERV1	1	872	(7335)	86	
1213	23.4	6.8	7.9	scaffold155_1,113,688-1,134,337	14388	14950	(5700)	+	ERV1-4-EC_I-int	LTR/ERV1	826	1305	(7178)	87	*
1385	21.2	10.2	6.7	scaffold155_1,113,688-1,134,337	15011	15500	(5150)	C	CarLTR1B2	LTR/ERV1	(40)	506	1	88	
1302	24.5	4.6	4.0	scaffold155_1,113,688-1,134,337	15501	15938	(4712)	+	MER34-int	LTR/ERV1	920	1220	(5089)	86	
596	17.7	15.7	4.6	scaffold155_1,113,688-1,134,337	15961	16138	(4512)	C	SINEC1_AMe	SINE/tRNA	(4)	197	1	89	
686	21.7	16.2	2.0	scaffold155_1,113,688-1,134,337	16189	16404	(4246)	+	MER34-int	LTR/ERV1	2156	2401	(6082)	86	
759	23.5	9.5	1.8	scaffold155_1,113,688-1,134,337	16469	16529	(4121)	+	MER34-int	LTR/ERV1	3020	3093	(4170)	86	
649	11.6	0.0	0.0	scaffold155_1,113,688-1,134,337	16530	16624	(4026)	+	SINEC_old	SINE/tRNA	1	95	(17)	90	

759	23.5	9.5	1.8	scaffold155_1,113,688-1,134,337	16625	16826	(3824)	+	ERV54-EC_I-int	LTR/ERV1	3094	3303	(3960)	91	
2648	11.1	1.3	0.2	scaffold155_1,113,688-1,134,337	17416	17804	(2846)	+	LTR31_AMe	LTR/ERV1	1	393	(131)	92	
303	13.7	1.9	1.9	scaffold155_1,113,688-1,134,337	17804	17855	(2795)	C	SINEC_old	SINE/tRNA	(58)	54	3	93	*
12	13.9	0.0	0.0	scaffold155_1,113,688-1,134,337	17856	17879	(2771)	+	(ACC)n	Simple_repeat	1	24	(0)	94	
1367	18.8	5.0	1.4	scaffold155_1,113,688-1,134,337	17923	18263	(2387)	+	ERV54-EC_I-int	LTR/ERV1	3704	4056	(3207)	91	
641	15.2	2.0	0.0	scaffold155_1,113,688-1,134,337	18289	18387	(2263)	+	SINEC_old	SINE/tRNA	9	109	(3)	95	
351	23.8	0.0	5.0	scaffold155_1,113,688-1,134,337	18396	18501	(2149)	+	MER34-int	LTR/ERV1	3187	3287	(2903)	86	*
484	18.3	0.0	0.0	scaffold155_1,113,688-1,134,337	18408	18505	(2145)	+	ERV1-5N-EC_I-int	LTR/ERV1	2272	2357	(3254)	96	
486	12.2	21.4	0.8	scaffold155_1,113,688-1,134,337	18508	18607	(2043)	C	SINEC_b1	SINE/tRNA	(125)	103	1	97	
1592	23.5	9.7	10.1	scaffold155_1,113,688-1,134,337	18628	19792	(858)	+	MER34-int	LTR/ERV1	4243	5403	(1860)	86	
332	17.2	5.2	0.0	scaffold155_1,113,688-1,134,337	19808	19865	(785)	+	SINEC_old	SINE/tRNA	34	94	(18)	98	
1103	19.0	9.9	6.8	scaffold155_1,113,688-1,134,337	19867	20090	(560)	+	MER34-int	LTR/ERV1	5398	5638	(1625)	86	
1633	7.5	0.0	0.0	scaffold155_1,113,688-1,134,337	20091	20291	(359)	C	SINEC1_AMe	SINE/tRNA	(0)	201	1	99	
1103	19.3	10.7	7.1	scaffold155_1,113,688-1,134,337	20292	20498	(152)	+	MER34-int	LTR/ERV1	5639	5791	(1472)	86	
241	27.9	3.6	9.2	scaffold155_1,113,688-1,134,337	20499	20636	(14)	+	HAL1-1D_Tbel	LINE/L1	1785	1915	(615)	100	
2352	6.4	0.0	0.0	scaffold162_1-18,384_(reversed)	1	295	(18089)	+	LSU-rRNA_Hsa	rRNA	2420	2714	(2321)	101	
313	15.9	8.8	10.0	scaffold162_1-18,384_(reversed)	296	386	(17998)	C	ERV3-1_SSc-LTR	LTR/ERV1	(312)	137	48	102	
1371	0.0	0.0	1.2	scaffold162_1-18,384_(reversed)	387	558	(17826)	+	LSU-rRNA_Hsa	rRNA	2806	2975	(2060)	103	
12353	3.9	1.2	0.8	scaffold162_1-18,384_(reversed)	765	2291	(16093)	+	LSU-rRNA_Hsa	rRNA	3502	5035	(0)	103	
38	4.7	0.0	0.0	scaffold162_1-18,384_(reversed)	3012	3055	(15329)	+	(GTG)n	Simple_repeat	1	44	(0)	104	
313	20.0	2.8	1.4	scaffold162_1-18,384_(reversed)	3070	3140	(15244)	C	tRNA-Lys-AAG	tRNA	(3)	73	2	105	
253	17.8	0.0	2.2	scaffold162_1-18,384_(reversed)	3827	3872	(14512)	C	CarLTR1-int	LTR/ERV1	(543)	4844	4800	106	*
266	12.2	0.0	0.0	scaffold162_1-18,384_(reversed)	3871	3911	(14473)	C	CarLTR1-int	LTR/ERV1	(765)	4622	4582	106	
69	6.9	1.7	4.3	scaffold162_1-18,384_(reversed)	3923	4040	(14344)	+	(TCTT)n	Simple_repeat	1	115	(0)	107	
492	18.0	3.0	1.0	scaffold162_1-18,384_(reversed)	4041	4141	(14243)	C	CarLTR1-int	LTR/ERV1	(2683)	3932	3830	106	
612	9.9	0.0	0.0	scaffold162_1-18,384_(reversed)	4143	4223	(14161)	C	L1_Canid_	LINE/L1	(1120)	5331	5251	108	
483	29.1	2.2	9.7	scaffold162_1-18,384_(reversed)	4225	4589	(13795)	C	Sat-1_TSy	Satellite	(1106)	2130	1791	109	
2686	22.4	2.9	3.4	scaffold162_1-18,384_(reversed)	4646	5298	(13086)	+	ERV1-4N-EC_I-int	LTR/ERV1	5486	6135	(174)	110	
672	10.6	1.1	0.0	scaffold162_1-18,384_(reversed)	5381	5474	(12910)	+	SINEC_old	SINE/tRNA	1	95	(17)	111	
503	22.5	3.7	4.0	scaffold162_1-18,384_(reversed)	7462	7643	(10741)	+	LTRIS4	LTR/ERV1	309	465	(42)	112	
713	31.9	0.7	1.7	scaffold162_1-18,384_(reversed)	8196	8485	(9899)	+	KORV_I-int	LTR/ERV1	457	743	(6681)	113	
2610	27.4	3.9	2.8	scaffold162_1-18,384_(reversed)	8736	10001	(8383)	+	KORV_I-int	LTR/ERV1	1024	2334	(5090)	113	
2263	31.2	5.3	6.8	scaffold162_1-18,384_(reversed)	11487	13001	(5383)	+	KORV_I-int	LTR/ERV1	4189	5682	(1742)	113	
360	35.2	0.0	0.0	scaffold162_1-18,384_(reversed)	13060	13201	(5183)	+	KORV_I-int	LTR/ERV1	5813	5954	(1470)	113	
291	28.4	0.0	0.0	scaffold162_1-18,384_(reversed)	13240	13313	(5071)	+	KORV_I-int	LTR/ERV1	6023	6096	(1328)	113	
891	33.4	3.0	2.6	scaffold162_1-18,384_(reversed)	13361	13821	(4563)	+	KORV_I-int	LTR/ERV1	6962	7424	(0)	113	
503	22.3	4.7	3.4	scaffold162_1-18,384_(reversed)	14243	14448	(3936)	+	LTRIS4	LTR/ERV1	205	465	(42)	114	
1601	9.7	5.8	0.0	scaffold162_1-18,384_(reversed)	14887	15144	(3240)	C	L1-1_AMe	LINE/L1	(4)	6843	6571	115	
1233	29.7	6.2	2.7	scaffold182_1,642,082-1,661,437	412	1156	(18200)	+	L1M5	LINE/L1	4413	5182	(964)	116	
295	30.7	8.9	2.7	scaffold182_1,642,082-1,661,437	1198	1445	(17911)	+	L1M5	LINE/L1	5254	5516	(607)	116	
289	17.7	3.2	1.6	scaffold182_1,642,082-1,661,437	1461	1523	(17833)	+	L1M5	LINE/L1	5573	5636	(487)	116	
1242	18.8	4.4	1.9	scaffold182_1,642,082-1,661,437	1583	1856	(17500)	+	LTR22_FC	LTR/ERV1	229	489	(0)	117	
287	25.1	14.4	1.8	scaffold182_1,642,082-1,661,437	1867	2012	(17344)	+	L1ME3E	LINE/L1	5780	5943	(299)	118	
421	32.8	5.0	4.7	scaffold182_1,642,082-1,661,437	2232	2589	(16767)	+	ERVL-E-int	LTR/ERVL	2725	3083	(2584)	119	
1114	30.3	5.9	2.8	scaffold182_1,642,082-1,661,437	2759	3253	(16103)	+	ERVL-E-int	LTR/ERVL	3307	3816	(1851)	119	
273	31.5	1.6	4.9	scaffold182_1,642,082-1,661,437	3452	3578	(15778)	+	ERVL-E-int	LTR/ERVL	4169	4291	(1470)	119	
20	26.5	3.5	5.4	scaffold182_1,642,082-1,661,437	3631	3744	(15612)	+	A-rich	Low_complexity	1	112	(0)	120	
266	8.3	10.4	0.0	scaffold182_1,642,082-1,661,437	4050	4097	(15259)	C	MLT2B1	LTR/ERVL	(3)	564	512	121	
234	28.1	1.8	0.0	scaffold182_1,642,082-1,661,437	4138	4194	(15162)	C	HAL1-3A_ME	LINE/L1	(835)	2023	1966	122	
530	12.3	0.0	0.0	scaffold182_1,642,082-1,661,437	4457	4537	(14819)	C	L1_Carn5	LINE/L1	(8)	6501	6421	123	
11	24.3	0.0	0.0	scaffold182_1,642,082-1,661,437	4546	4579	(14777)	+	(TTTTAT)n	Simple_repeat	1	34	(0)	124	
383	25.7	0.0	2.9	scaffold182_1,642,082-1,661,437	4589	4696	(14660)	+	MLT1H	LTR/ERVL-MaLR	5	109	(440)	125	
1174	14.0	0.0	5.5	scaffold182_1,642,082-1,661,437	5380	5591	(13765)	C	SINEC1_AMe	SINE/tRNA	(0)	201	1	126	
1550	20.0	0.3	8.0	scaffold182_1,642,082-1,661,437	5596	6003	(13353)	C	L1MB3_EC	LINE/L1	(624)	5590	5220	127	
1479	24.2	2.7	2.7	scaffold182_1,642,082-1,661,437	6004	6445	(12911)	+	L1ME1	LINE/L1	5505	5946	(233)	128	
366	27.0	2.7	0.0	scaffold182_1,642,082-1,661,437	6475	6585	(12771)	+	L1ME1	LINE/L1	6044	6157	(22)	128	
16	13.7	2.9	2.9	scaffold182_1,642,082-1,661,437	7203	7236	(12120)	+	(TA)n	Simple_repeat	1	34	(0)	129	
481	21.1	3.9	2.2	scaffold182_1,642,082-1,661,437	9787	9972	(9384)	+	LTRIS4	LTR/ERV1	144	462	(45)	130	
299	24.7	9.4	0.6	scaffold182_1,642,082-1,661,437	9996	10147	(9209)	C	MIR_Mars	SINE/MIR	(13)	261	96	131	
608	21.1	0.8	0.0	scaffold182_1,642,082-1,661,437	10637	10764	(8592)	+	L1-1_AMe	LINE/L1	6700	6828	(19)	132	
1366	8.6	0.5	0.0	scaffold182_1,642,082-1,661,437	10771	10957	(8399)	+	SINEC1_AMe	SINE/tRNA	1	188	(13)	133	
47	0.0	0.0	0.0	scaffold182_1,642,082-1,661,437	10961	11003	(8353)	+	(AAAT)n	Simple_repeat	1	43	(0)	134	
234	19.5	0.0	6.0	scaffold182_1,642,082-1,661,437	11062	11132	(8224)	+	LTR1A2_ML	LTR/ERV1	510	576	(191)	135	
326	31.4	8.8	1.4	scaffold182_1,642,082-1,661,437	11452	11793	(7563)	+	L1ME3F	LINE/L1	4303	4669	(1477)	136	
454	23.7	2.5	8.8	scaffold182_1,642,082-1,661,437	12150	12390	(6966)	C	SINEC1B_AMe	SINE/tRNA	(0)	228	2	137	
265	26.2	3.9	3.4	scaffold182_1,642,082-1,661,437	12565	12742	(6614)	+	L1ME3F	LINE/L1	5264	5442	(801)	136	*
293	27.0	1.2	2.4	scaffold182_1,642,082-1,661,437	12629	12771	(6585)	+	L1P5	LINE/L1	5329	5453	(701)	138	*
1147	24.8	9.6	2.4	scaffold182_1,642,082-1,661,437	12770	13206	(6150)	+	L1ME3F	LINE/L1	5494	5961	(282)	136	
564	22.9	3.4	0.0	scaffold182_1,642,082-1,661,437	14452	14569	(4787)	+	SINEC2_AMe	SINE/tRNA	1	122	(11)	139	
338	8.7	14.5	0.0	scaffold182_1,642,082-1,661,437	14574	14642	(4714)	+	SINEC_c2	SINE/tRNA	132	210	(2)	140	
16	21.6	2.3	0.0	scaffold182_1,642,082-1,661,437	14714	14756	(4600)	+	A-rich	Low_complexity	1	44	(0)	141	
243	36.5	3.3	2.2	scaffold182_1,642,082-1,661,437	15286	15467	(3889)	+	L2-2_ME	LINE/L2	1655	1838	(703)	142	
23	24.5	1.4	1.4	scaffold182_1,642,082-1,661,437	16631	16700	(2656)	+	GA-rich	Low_complexity	1	70	(0)	143	
694	20.8	1.3	0.7	scaffold182_1,642,082-1,661,437	17549	17698	(1658)	C	L1_Canid_	LINE/L1	(0)	6451	6301	144	
533	23.2	6.3	9.6	scaffold182_1,642,082-1,661,437	18645	18849	(507)	+	SINEC1_AMe	SINE/tRNA	1	199	(2)	145	
24	19.8	1.5	3.0	scaffold182_1,642,082-1,661,437	19197	19264	(92)	+	(ATAGAT)n	Simple_repeat	1	66	(0)	146	
1553	7.1	0.5	0.0	scaffold1_66,687,907-66,667,100_(reversed)	67	262	(20546)	+	SINEC1_AMe	SINE/tRNA	1	197	(4)	147	
592	29.5	1.7	7.5	scaffold1_66,687,907-66,667,100_(reversed)	1100	1188	(19620)	+	L1MC1	LINE/L1	4142	4529	(1905)	148	
1784	18.4	0.0	0.6	scaffold1_66,687,907-66,667,100_(reversed)	1189	1506	(19302)	+	L1MC	LINE/L1	3984	4299	(1847)	149	
812	12.3	28.4	0.0	scaffold1_66,687,907-66,667,100_(reversed)	1507	1661	(19147)	+	SINEC_c1	SINE/tRNA	1	199	(10)	150	
592	29.5	1.7	7.5	scaffold1_66,687,907-66,667,100_(reversed)	1662	1675	(19133)	+	L1MC	LINE/L1	4530	4540	(1894)	149	
3465	22.0	2.2	4.5	scaffold1_66,687,907-66,667,100_(reversed)	1688	2859	(17949)	+	L1MC1	LINE/L1	4288	6277	(723)	148	*
4265	15.6	0.9	2.5	scaffold1_66,687,907-66,667,100_(reversed)	2075	2861	(17947)	+	L1MC1_EC	LINE/L1	4674	5448	(897)	151	
823	13.5	0.8	0.0	scaffold1_66,687,907-66,667,100_(reversed)	2869	2994	(17814)	+	SINEC_b1	SINE/tRNA	1	127	(67)	152	
29	0.0	0.0	0.0	scaffold1_66,687,907-66,667,100_(reversed)	2995	3019	(17789)	+	(TC)n	Simple_repeat	1	25	(0)	153	
4560	18.5	3.9	0.0	scaffold1_66,687,907-66,667,100_(reversed)	3056	3932	(16876)	+	L1MC1	LINE/L1	5423	6333	(0)	148	
433	30.9	2.1	4.9	scaffold1_66,687,907-66,667,100_(reversed)	4808	5032	(15776)	C	MamGypLTR3	LTR/Gypsy	(18)	963	711	154	
269	33.7	2.4	0.0	scaffold1_66,687,907-66,667,100_(reversed)	5347	5512	(15296)	C	MamGypLTR3	LTR/Gypsy	(609)	285	66	154	
231	27.7	10.8	0.9	scaffold1_66,687,907-66,667,100_(reversed)	5842	5943	(14865)	C	MIR3_MarsB	SINE/MIR	(71)	144	33	155	
513	21.7	4.7	3.4	scaffold1_66,687,907-66,667,100_(reversed)	6386	6591	(14217)	+	LTRIS4	LTR/ERV1	205	465	(42)	156	
253	28.4	0.0	1.1	scaffold1_66,687,907-66,667,100_(reversed)	6842	6930	(13878)	+	KORV_I-int	LTR/ERV1	198	285	(7297)	157	
13	20.6	2.4	2.4	scaffold1_66,687,907-66,667,100_(reversed)	7071	7111	(13697)	+	(TGTT)n	Simple_repeat	1	41	(0)	158	
754	30.9	0.7	1.7	scaffold1_66,687,907-66,667,100_(reversed)	7143	7432	(13376)	+	KORV_I-int	LTR/ERV1	457	743	(6681)	157	
6139	29.3	2.8	2.5	scaffold1_66,687,907-66,667,100_(reversed)	7683	10804	(10004)	+	KORV_I-int	LTR/ERV1	1024	4183	(3241)	157	
269	33.7	2.4	0.0	scaffold1_66,687,907-66,667,100_(reversed)	12340	12505	(8303)	C	MamGypLTR3	LTR/Gypsy	(609)	285	66	159	
231	27.7	10.8	0.9	scaffold1_66,687,907-66,667,100_(reversed)	12835	12936	(7872)	C	MIR3_MarsB	SINE/MIR	(71)	144	33	160	
513	21.7	4.7	3.4	scaffold1_66,687,907-66,667,100_(reversed)	13379	13584	(7224)	+	LTRIS4	LTR/ERV1	205	465	(42)	161	
238	26.9	1.0	5.3	scaffold1_66,687,907-66,667,100_(reversed)	13628	13725	(7083)	C	Mon1f4	SINE/MIR	(120)	147	54	162	
220	29.4	2.6	3.9	scaffold1_66,687,907-66,667,100_(reversed)	14406	14483	(6325)	C	MIRb	SINE/MIR	(26)	242	166	163	
234	31.2	2.1	0.0	scaffold1_66,687,907-66,667,100_(reversed)	14860	14955	(5853)	+	MIR3	SINE/MIR	111	208	(0)	164	
32	23.4	0.0	0.0	scaffold1_66,687,907-66,667,100_(reversed)	16909	16978	(3830)	+	A-rich	Low_complexity	1	70	(0)	165	
31	19.6	4.8	1.2	scaffold1_66,687,907-66,667,100_(reversed)	17536	17618	(3190)	+	(AT)n	Simple_repeat	1	86	(0)	166	
217	27.7	14.7	0.6	scaffold1_66,687,907-66,667,100_(reversed)	17665	17820	(2988)	C	MIRc	SINE/MIR	(27)	241	64	167	
485	24.5	0.0	0.9	scaffold1_66,687,907-66,667,100_(reversed)	17910	18020	(2788)	+	ORSL	DNA/hAT-Tip100	1	110	(165)	168	*
607	14.5	10.3	2.9	scaffold1_66,687,907-66,667,100_(reversed)	18015	18146	(2662)	+	SINEC_c2	SINE/tRNA	75	219	(0)	169	
23	22.7	2.7	2.7	scaffold1_66,687,907-66,667,100_(reversed)	18216	18288	(2520)	+	G-rich	Low_complexity	1	73	(0)	170	
271	27.5	0.0	0.0	scaffold1_66,687,907-66,667,100_(reversed)	18340	18408	(2400)	+	L2c	LINE/L2	3091	3159	(228)	171	
1634	20.4	5.8	1.2	scaffold1_66,687,907-66,667,100_(reversed)	19129	19271	(1537)	C	L1MA9	LINE/L1	(0)	6312	6167	172	
36	0.0	0.0	0.0	scaffold1_66,687,907-66,667,100_(reversed)	19272	19302	(1506)	+	(GT)n	Simple_repeat	1	31	(0)	173	
1634	20.4	5.8	1.2	scaffold1_66,687,907-66,667,100_(reversed)	19303	19540	(1268)	C	L1MA9	LINE/L1	(146)	6166	5915	172	
25	10.0	2.3	0.0	scaffold1_66,687,907-66,667,100_(reversed)	20157	20199	(609)	+	(TTTGT)n	Simple_repeat	1	44	(0)	174	
55	1.7	3.3	0.0	scaffold1_66,687,907-66,667,100_(reversed)	20513	20573	(235)	+	(TTAT)n	Simple_repeat	1	63	(0)	175	
264	16.3	2.6	3.4	scaffold200_144,464-153,663	6	79	(9121)	+	L1_Mur2	LINE/L1	2903	2958	(2919)	176	
510	29.6	10.2	3.2	scaffold200_144,464-153,663	120	590	(8610)	+	ERVL-E-int	LTR/ERVL	1262	1790	(3924)	177	
503	22.3	4.7	3.4	scaffold200_144,464-153,663	1021	1226	(7974)	+	LTRIS4	LTR/ERV1	205	465	(42)	178	
743	31.2	0.7	1.7	scaffold200_144,464-153,663	1378	1667	(7533)	+	KORV_I-int	LTR/ERV1	457	743	(6681)	179	
6735	29.6	3.1	3.4	scaffold200_144,464-153,663	1918	6560	(2640)	+	KORV_I-int	LTR/ERV1	1024	5682	(1742)	179	
240	35.0	0.0	0.0	scaffold200_144,464-153,663	6658	6760	(2440)	+	KORV_I-int	LTR/ERV1	5852	5954	(1470)	179	
276	22.6	0.0	0.0	scaffold200_144,464-153,663	6820	6872	(2328)	+	KORV_I-int	LTR/ERV1	6044	6096	(1328)	179	
383	36.2	2.5	2.1	scaffold200_144,464-153,663	7056	7292	(1908)	+	KORV_I-int	LTR/ERV1	6300	6537	(887)	179	
1293	33.3	3.6	4.2	scaffold200_144,464-153,663	7412	8217	(983)	+	KORV_I-int	LTR/ERV1	6624	7424	(0)	179	
476	22.4	6.5	2.3	scaffold200_144,464-153,663	8654	8833	(367)	+	LTRIS4	LTR/ERV1	205	465	(42)	180	

341	30.6	13.4	1.2	scaffold200_144,464-153,663	8956	9104	(96)	+	MER63A	DNA/hAT-Blackjack	7	173	(37)	181	
31	2.5	2.4	2.4	scaffold200_144,464-153,663	9137	9178	(22)	+	(TCTT)n	Simple_repeat	1	42	(0)	182	
1687	11.0	2.5	0.0	scaffold211_1,354,903-1,360,362	235	470	(4990)	+	CarLTR1-int	LTR/ERV1	1319	1560	(5055)	183	
311	9.8	0.0	0.0	scaffold211_1,354,903-1,360,362	473	513	(4947)	+	ERV54-EC_I-int	LTR/ERV1	4000	4040	(3223)	184	*
726	7.5	33.3	1.1	scaffold211_1,354,903-1,360,362	497	688	(4772)	+	CarLTR1-int	LTR/ERV1	1993	2248	(4367)	183	
581	6.7	0.0	0.0	scaffold211_1,354,903-1,360,362	688	762	(4698)	+	HUERS-P3-int	LTR/ERV1	2007	2081	(4534)	185	*
1855	33.5	4.4	2.5	scaffold211_1,354,903-1,360,362	915	1953	(3507)	+	HUERS-P3-int	LTR/ERV1	2634	3691	(5228)	185	
374	32.9	8.2	2.9	scaffold211_1,354,903-1,360,362	1954	2247	(3213)	+	HUERS-P3-int	LTR/ERV1	2746	3054	(4370)	186	
755	34.3	0.4	0.0	scaffold211_1,354,903-1,360,362	2251	2515	(2945)	+	HUERS-P3-int	LTR/ERV1	3977	4242	(2849)	186	
3401	32.2	2.8	1.7	scaffold211_1,354,903-1,360,362	2530	3910	(1550)	+	HUERS-P3-int	LTR/ERV1	4367	5762	(3157)	186	
481	33.9	5.6	3.4	scaffold211_1,354,903-1,360,362	3916	4288	(1172)	+	KORV_I-int	LTR/ERV1	4741	5121	(2303)	187	
375	30.0	2.4	2.4	scaffold211_1,354,903-1,360,362	4405	4568	(892)	+	HUERS-P3-int	LTR/ERV1	6652	6815	(1811)	186	
679	27.2	4.6	4.6	scaffold211_1,354,903-1,360,362	4673	4888	(572)	+	LTR77-int_TS	LTR/ERV1	5336	5551	(0)	188	
1071	20.4	2.9	7.1	scaffold211_1,354,903-1,360,362	5009	5287	(173)	+	CarLTR9	LTR/ERV1	296	563	(0)	189	
641	23.7	3.5	0.0	scaffold211_1,354,903-1,360,362	5288	5460	(0)	C	L1MAB_EC	LINE/L1	(106)	6788	6610	190	
784	19.0	7.1	2.1	scaffold217_224,999-245,612	1	183	(20431)	+	SINEC_c2	SINE/tRNA	21	212	(0)	191	
723	17.1	10.6	3.5	scaffold217_224,999-245,612	214	401	(20213)	C	SINEC1_AMe	SINE/tRNA	(0)	201	1	192	
17	29.7	0.0	0.0	scaffold217_224,999-245,612	2257	2305	(18309)	+	(AC)n	Simple_repeat	1	49	(0)	193	
1008	8.8	16.7	8.4	scaffold217_224,999-245,612	2325	2534	(18080)	+	SINEC1B_AMe	SINE/tRNA	1	226	(2)	194	
225	11.8	0.0	0.0	scaffold217_224,999-245,612	2659	2692	(17922)	C	ORR1A3-int	LTR/ERVL-MaLR	(11)	1952	1919	195	
189	29.2	5.6	1.1	scaffold217_224,999-245,612	2804	2893	(17721)	+	MIR	SINE/MIR	166	259	(3)	196	
40	0.0	0.0	0.0	scaffold217_224,999-245,612	3324	3357	(17257)	+	(CA)n	Simple_repeat	1	34	(0)	197	
222	23.1	18.2	0.0	scaffold217_224,999-245,612	4112	4254	(16360)	C	MIR	SINE/MIR	(53)	215	40	198	
269	27.1	1.0	2.1	scaffold217_224,999-245,612	4350	4447	(16167)	C	WALLSI4	SINE/tRNA-Core-RTE	(108)	141	45	199	*
281	26.9	4.0	4.1	scaffold217_224,999-245,612	4360	4460	(16154)	C	Mon1g3	SINE/MIR	(153)	134	38	200	
196	26.1	0.0	7.8	scaffold217_224,999-245,612	5340	5422	(15192)	+	L2d	LINE/L2	3018	3094	(370)	201	
21	18.4	0.0	9.0	scaffold217_224,999-245,612	7660	7732	(12882)	+	(TCTAGAA)n	Simple_repeat	1	67	(0)	202	
283	27.4	2.4	1.2	scaffold217_224,999-245,612	7989	8073	(12541)	C	MIRb	SINE/MIR	(68)	200	115	203	
315	29.4	6.5	0.9	scaffold217_224,999-245,612	9252	9450	(11164)	+	MIR	SINE/MIR	12	221	(41)	204	
367	26.3	2.1	9.0	scaffold217_224,999-245,612	9728	9869	(10745)	C	PlatSat2A	Satellite	(642)	391	259	205	
502	21.9	14.6	3.8	scaffold217_224,999-245,612	10045	10221	(10393)	C	LTRIS4	LTR/ERV1	(39)	468	309	206	
199	26.1	0.0	0.0	scaffold217_224,999-245,612	10726	10771	(9843)	C	MIR3	SINE/MIR	(61)	147	102	207	
28	21.0	1.6	0.0	scaffold217_224,999-245,612	11496	11556	(9058)	+	(CT)n	Simple_repeat	1	62	(0)	208	
15	15.5	2.8	0.0	scaffold217_224,999-245,612	11623	11658	(8956)	+	(CTCCTTC)n	Simple_repeat	1	37	(0)	209	
15	13.2	4.4	4.4	scaffold217_224,999-245,612	11823	11867	(8747)	+	(TCCTCAC)n	Simple_repeat	1	45	(0)	210	
348	27.9	7.2	4.4	scaffold217_224,999-245,612	13194	13635	(6979)	+	L2b	LINE/L2	2921	3374	(1)	211	
255	22.9	0.0	1.4	scaffold217_224,999-245,612	14017	14087	(6527)	+	MSR1	Satellite	10	79	(32)	212	
36	17.5	0.0	1.4	scaffold217_224,999-245,612	14332	14405	(6209)	+	G-rich	Low_complexity	1	73	(0)	213	
325	31.9	4.2	1.0	scaffold217_224,999-245,612	14572	14764	(5850)	+	L1ME5	LINE/L1	5661	5859	(335)	214	
443	19.8	3.8	4.8	scaffold217_224,999-245,612	14913	15018	(5596)	C	MER77	LTR/ERVL	(0)	604	500	215	
1399	24.0	5.9	4.6	scaffold217_224,999-245,612	15039	15462	(5152)	C	MER77	LTR/ERVL	(72)	532	105	215	
259	15.4	0.0	0.0	scaffold217_224,999-245,612	15484	15522	(5092)	C	L2B_ME	LINE/L2	(18)	3429	3391	216	
13	4.7	0.0	4.3	scaffold217_224,999-245,612	15705	15728	(4886)	+	(GAGG)n	Simple_repeat	1	23	(0)	217	
606	31.6	7.4	0.9	scaffold217_224,999-245,612	15800	16421	(4193)	+	L1M5	LINE/L1	5254	5703	(491)	218	
1424	12.0	0.5	0.0	scaffold217_224,999-245,612	16565	16764	(3850)	+	SINEC1_AMe	SINE/tRNA	1	201	(0)	219	
13	27.7	4.9	0.0	scaffold217_224,999-245,612	17301	17361	(3253)	+	(CTCTGT)n	Simple_repeat	1	64	(0)	220	
1386	18.6	7.9	4.3	scaffold217_224,999-245,612	17616	18020	(2594)	C	L1MC	LINE/L1	(1117)	5029	4611	221	
2237	17.4	2.7	2.3	scaffold217_224,999-245,612	18099	18459	(2155)	C	L1MC	LINE/L1	(1489)	4657	4291	221	
2268	16.0	4.5	3.1	scaffold217_224,999-245,612	18460	18905	(1709)	+	CarERV4a_LTR	LTR/ERV1	5	456	(0)	222	
2237	17.4	2.7	2.3	scaffold217_224,999-245,612	18906	19018	(1596)	C	L1MC	LINE/L1	(1856)	4290	4181	221	
1580	7.0	0.0	1.0	scaffold217_224,999-245,612	19024	19226	(1388)	C	SINEC1_AMe	SINE/tRNA	(0)	201	1	223	
727	23.0	2.1	8.7	scaffold217_224,999-245,612	19231	19463	(1151)	C	L1MC1_EC	LINE/L1	(2144)	4201	3983	224	
300	30.4	8.4	3.3	scaffold217_224,999-245,612	19470	19612	(1002)	C	LTR40a	LTR/ERVL	(219)	300	151	225	
989	12.8	6.4	7.0	scaffold217_224,999-245,612	20102	20303	(311)	+	SINEC1_AMe	SINE/tRNA	1	201	(0)	226	
218	25.3	11.4	0.0	scaffold30_5,823,614-5,844,139	348	426	(20100)	C	L2d	LINE/L2	(86)	3339	3252	227	
424	28.1	7.1	6.7	scaffold30_5,823,614-5,844,139	747	998	(19528)	+	MamRep605	LTR?	210	462	(387)	228	
773	25.9	1.6	3.2	scaffold30_5,823,614-5,844,139	1130	1384	(19142)	C	MIR	SINE/MIR	(10)	252	2	229	
325	23.9	2.0	0.0	scaffold30_5,823,614-5,844,139	1863	1962	(18564)	+	L1_Canid_	LINE/L1	6364	6451	(0)	230	
297	31.5	8.1	10.3	scaffold30_5,823,614-5,844,139	3135	3481	(17045)	+	L1M6	LINE/L1	682	1021	(5475)	231	
404	30.6	9.8	0.4	scaffold30_5,823,614-5,844,139	3648	3893	(16633)	+	L1M6	LINE/L1	1251	1519	(4977)	231	
244	24.4	0.0	1.9	scaffold30_5,823,614-5,844,139	4133	4222	(16304)	+	L1M6	LINE/L1	1813	1988	(688)	231	
1061	8.7	2.0	0.0	scaffold30_5,823,614-5,844,139	4770	4918	(15608)	+	L1_Carn3	LINE/L1	6285	6436	(1)	232	
19	13.7	2.5	0.0	scaffold30_5,823,614-5,844,139	4935	4974	(15552)	+	A-rich	Low_complexity	1	41	(0)	233	
361	23.5	16.9	3.6	scaffold30_5,823,614-5,844,139	5041	5212	(15314)	C	SINEC1_AMe	SINE/tRNA	(7)	194	1	234	
1606	5.5	1.0	0.0	scaffold30_5,823,614-5,844,139	5427	5625	(14901)	C	SINEC1_AMe	SINE/tRNA	(0)	201	1	235	
24	20.9	0.0	0.0	scaffold30_5,823,614-5,844,139	5850	5904	(14622)	+	(AATG)n	Simple_repeat	1	55	(0)	236	
12	20.2	2.9	0.0	scaffold30_5,823,614-5,844,139	6136	6169	(14357)	+	(ATATA)n	Simple_repeat	1	35	(0)	237	
488	25.8	0.8	0.0	scaffold30_5,823,614-5,844,139	6220	6339	(14187)	+	SINEC2_AMe	SINE/tRNA	4	124	(9)	238	
356	13.9	15.1	0.0	scaffold30_5,823,614-5,844,139	6340	6425	(14101)	+	SINEC_Fc3	SINE/tRNA	128	226	(1)	239	
387	33.9	1.6	0.0	scaffold30_5,823,614-5,844,139	7383	7509	(13017)	+	MIRb	SINE/MIR	44	172	(96)	240	
565	20.3	0.8	0.0	scaffold30_5,823,614-5,844,139	8747	8869	(11657)	+	SINEC2_AMe	SINE/tRNA	1	124	(9)	241	
242	19.5	6.4	1.2	scaffold30_5,823,614-5,844,139	8871	8948	(11578)	+	SINEC_c1	SINE/tRNA	126	207	(2)	242	
303	36.0	7.5	1.9	scaffold30_5,823,614-5,844,139	9735	9989	(10537)	C	L2c	LINE/L2	(85)	3302	3034	243	
513	22.1	4.1	4.0	scaffold30_5,823,614-5,844,139	10010	10215	(10311)	C	LTRIS4	LTR/ERV1	(42)	465	205	244	
378	18.5	18.4	0.8	scaffold30_5,823,614-5,844,139	10698	10806	(9720)	+	MER96B	DNA/hAT-Tip100	49	176	(241)	245	
53	1.9	0.0	0.0	scaffold30_5,823,614-5,844,139	10994	11045	(9481)	+	(TTA)n	Simple_repeat	1	52	(0)	246	
489	14.6	7.7	2.3	scaffold30_5,823,614-5,844,139	11095	11257	(9269)	+	SINEC_a2	SINE/tRNA	1	179	(0)	247	
12	8.9	4.2	0.0	scaffold30_5,823,614-5,844,139	11269	11292	(9234)	+	(TTTA)n	Simple_repeat	1	25	(0)	248	
658	16.5	26.7	2.0	scaffold30_5,823,614-5,844,139	11297	11457	(9069)	C	SINEC_c1	SINE/tRNA	(9)	200	1	249	
240	30.3	9.0	9.0	scaffold30_5,823,614-5,844,139	11545	11722	(8804)	+	MIRc	SINE/MIR	51	231	(37)	250	
782	18.5	11.9	3.5	scaffold30_5,823,614-5,844,139	12219	12403	(8123)	+	SINEC1_AMe	SINE/tRNA	2	201	(0)	251	
14	4.3	4.2	0.0	scaffold30_5,823,614-5,844,139	12894	12917	(7609)	+	(CATT)n	Simple_repeat	1	25	(0)	252	
442	9.5	1.2	1.3	scaffold30_5,823,614-5,844,139	13517	13598	(6928)	+	L1-1_AMe	LINE/L1	6162	6216	(631)	253	
1393	8.6	0.0	0.5	scaffold30_5,823,614-5,844,139	13861	14058	(6468)	+	L1-1_AMe	LINE/L1	6651	6847	(0)	253	
62	0.0	4.5	0.0	scaffold30_5,823,614-5,844,139	14941	15006	(5520)	+	(TTTTC)n	Simple_repeat	1	69	(0)	254	
15	21.1	0.0	0.0	scaffold30_5,823,614-5,844,139	15036	15064	(5462)	+	GA-rich	Low_complexity	1	29	(0)	255	
532	21.9	0.8	0.0	scaffold30_5,823,614-5,844,139	15070	15188	(5338)	C	SINEC2_AMe	SINE/tRNA	(13)	120	1	256	
447	27.6	10.1	1.9	scaffold30_5,823,614-5,844,139	16110	16356	(4170)	+	LTR37B	LTR/ERV1	197	463	(5)	257	
325	31.4	4.7	1.5	scaffold30_5,823,614-5,844,139	16398	16555	(3971)	C	MIR	SINE/MIR	(0)	274	105	258	
781	21.1	7.3	2.1	scaffold30_5,823,614-5,844,139	16814	16992	(3534)	C	MER5A	DNA/hAT-Charlie	(0)	189	2	259	
2434	18.5	2.6	2.2	scaffold30_5,823,614-5,844,139	17002	17505	(3021)	C	Tigger5a_Perissod	DNA/TcMar-Tigger	(1)	506	1	260	
19	22.8	0.0	0.0	scaffold30_5,823,614-5,844,139	18507	18547	(1979)	+	(T)n	Simple_repeat	1	41	(0)	261	
1629	14.5	2.5	2.5	scaffold30_5,823,614-5,844,139	18620	18944	(1582)	C	L1-1_AMe	LINE/L1	(18)	6829	6505	262	
1519	20.0	2.5	3.7	scaffold30_5,823,614-5,844,139	19436	19793	(733)	C	L1MB8	LINE/L1	(0)	6178	5825	263	
404	32.9	6.8	2.7	scaffold30_5,823,614-5,844,139	19796	20420	(106)	C	Sat-1_TSy	Satellite	(306)	650	1	264	
379	19.2	0.0	0.0	scaffold30_5,823,614-5,844,139	20441	20524	(2)	C	L1MB3_EC	LINE/L1	(1055)	5159	5087	265	
343	27.9	2.8	3.6	scaffold42_16,713,922-16,693,294	642	782	(19877)	+	L4_C_Mam	LINE/RTE-X	4869	5008	(130)	266	
807	12.6	0.8	2.5	scaffold42_16,713,922-16,693,294	818	939	(19720)	+	SINEC2_AMe	SINE/tRNA	8	127	(6)	267	
691	12.8	30.2	0.0	scaffold42_16,713,922-16,693,294	980	1128	(19531)	+	SINEC_b2	SINE/tRNA	1	194	(0)	268	
15	19.8	0.0	4.8	scaffold42_16,713,922-16,693,294	1370	1413	(19246)	+	(TCCA)n	Simple_repeat	1	42	(0)	269	
243	36.0	1.8	0.0	scaffold42_16,713,922-16,693,294	1617	1730	(18929)	C	PlatSat2A	Satellite	(652)	381	266	270	
1184	25.7	3.1	3.6	scaffold42_16,713,922-16,693,294	1804	2245	(18414)	C	MLT1H1	LTR/ERVL-MaLR	(66)	483	12	271	
276	22.7	2.7	0.0	scaffold42_16,713,922-16,693,294	2273	2347	(18312)	+	MIR	SINE/MIR	186	262	(0)	272	
291	28.1	2.5	14.5	scaffold42_16,713,922-16,693,294	3171	3332	(17327)	C	MIRb	SINE/MIR	(31)	237	93	273	
226	39.1	1.3	3.9	scaffold42_16,713,922-16,693,294	3604	3760	(16899)	+	MIR3	SINE/MIR	35	187	(21)	274	
378	19.1	0.0	1.6	scaffold42_16,713,922-16,693,294	3775	3838	(16821)	C	MER91C	DNA/hAT-Tip100	(0)	140	78	275	
215	24.6	3.3	0.0	scaffold42_16,713,922-16,693,294	4411	4471	(16188)	C	L2a	LINE/L2	(0)	3426	3364	276	
281	33.1	10.5	8.2	scaffold42_16,713,922-16,693,294	4501	4776	(15883)	C	L2a	LINE/L2	(170)	3256	2975	276	
269	26.3	3.9	1.3	scaffold42_16,713,922-16,693,294	5404	5480	(15179)	+	Mon1g3	SINE/MIR	73	151	(136)	277	
402	25.0	4.0	0.0	scaffold42_16,713,922-16,693,294	5567	5690	(14969)	C	MIR	SINE/MIR	(25)	237	109	278	
374	30.8	5.1	7.4	scaffold42_16,713,922-16,693,294	6733	6967	(13692)	C	MLT1K	LTR/ERVL-MaLR	(0)	595	366	279	
233	13.0	12.9	1.3	scaffold42_16,713,922-16,693,294	7002	7071	(13588)	C	BTLTR1D2	LTR/ERVK	(218)	468	391	280	
585	28.1	9.2	1.4	scaffold42_16,713,922-16,693,294	7224	7429	(13230)	+	MER58A	DNA/hAT-Charlie	3	224	(0)	281	
337	29.4	7.1	5.1	scaffold42_16,713,922-16,693,294	8064	8217	(12442)	C	MIR	SINE/MIR	(26)	242	79	282	
938	22.3	15.5	13.3	scaffold42_16,713,922-16,693,294	8218	8631	(12028)	C	ERV3-16A3_LTR	LTR/ERVL	(0)	422	1	283	
337	33.3	4.1	2.5	scaffold42_16,713,922-16,693,294	8632	8707	(11952)	C	MIR	SINE/MIR	(184)	78	4	282	
20	8.9	0.0	5.3	scaffold42_16,713,922-16,693,294	8803	8842	(11817)	+	(TCAT)n	Simple_repeat	1	38	(0)	284	
451	3.6	0.0	0.0	scaffold42_16,713,922-16,693,294	9951	10006	(10653)	+	SINEC_b2	SINE/tRNA	1	56	(138)	285	
503	22.3	4.7	3.4	scaffold42_16,713,922-16,693,294	10443	10648	(10011)	+	LTRIS4	LTR/ERV1	205	465	(42)	286	
267	24.0	0.0	0.0	scaffold42_16,713,922-16,693,294	10653	10702	(9957)	+	SINEC_b1	SINE/tRNA	49	98	(96)	287	
2842	11.7	1.1	3.2	scaffold42_16,713,922-16,693,294	10715	11154	(9505)	C	CarLTR1B1	LTR/ERV1	(147)	432	2	288	
950	22.3	5.9	8.3	scaffold42_16,713,922-16,693,294	11384	11740	(8919)	+	LTR22_FC	LTR/ERV1	1	349	(140)	289	

313	28.6	12.1	0.6	scaffold42_16,713,922-16,693,294	11941	12081	(8578)	C	MER91A	DNA/hAT-Tip100	(4)	192	36	290	
255	17.1	0.0	0.5	scaffold42_16,713,922-16,693,294	12502	12565	(8094)	C	Charlie7b_Mars	DNA/hAT-Charlie	(148)	124	62	291	
361	18.2	0.0	0.0	scaffold42_16,713,922-16,693,294	13052	13117	(7542)	+	MIR3	SINE/MIR	124	189	(19)	292	
182	28.4	11.9	0.0	scaffold42_16,713,922-16,693,294	13365	13431	(7228)	+	MIR3	SINE/MIR	90	164	(44)	293	
1642	19.5	16.8	5.9	scaffold42_16,713,922-16,693,294	13600	14122	(6537)	+	CarLTR2	LTR/ERV1	2	578	(0)	294	
398	23.2	16.1	0.7	scaffold42_16,713,922-16,693,294	14146	14275	(6384)	C	LTR33	LTR/ERVL	(2)	513	364	295	
2480	17.6	4.8	9.0	scaffold42_16,713,922-16,693,294	14727	15057	(5602)	C	CarLTR2	LTR/ERV1	(0)	578	253	296	
2725	13.6	1.4	0.2	scaffold42_16,713,922-16,693,294	15058	15486	(5173)	C	CarERV2b2_LTR	LTR/ERV1	(0)	434	1	297	
2480	17.6	4.8	9.0	scaffold42_16,713,922-16,693,294	15487	15755	(4904)	C	CarLTR2	LTR/ERV1	(326)	252	1	296	
198	33.8	1.3	0.0	scaffold42_16,713,922-16,693,294	15756	15832	(4827)	+	MIRb	SINE/MIR	113	190	(78)	298	
259	30.8	0.0	0.0	scaffold42_16,713,922-16,693,294	16511	16575	(4084)	+	MIRb	SINE/MIR	122	186	(82)	299	
30	0.0	0.0	0.0	scaffold42_16,713,922-16,693,294	16913	16938	(3721)	+	(AC)n	Simple_repeat	1	26	(0)	300	
18	33.9	0.0	0.0	scaffold42_16,713,922-16,693,294	16940	16994	(3665)	+	(CA)n	Simple_repeat	1	55	(0)	301	
339	27.5	1.1	0.0	scaffold42_16,713,922-16,693,294	17165	17255	(3404)	+	Mon1g1	SINE/MIR	59	150	(153)	302	
915	25.4	3.8	2.8	scaffold42_16,713,922-16,693,294	17720	18006	(2653)	+	L1ME3A	LINE/L1	5861	6150	(23)	303	
270	21.0	7.4	0.0	scaffold42_16,713,922-16,693,294	18129	18209	(2450)	C	MIRb	SINE/MIR	(82)	186	100	304	
1710	5.0	0.0	0.0	scaffold42_16,713,922-16,693,294	18300	18500	(2159)	+	SINEC1_AMe	SINE/tRNA	1	201	(0)	305	
14	4.3	0.0	7.7	scaffold42_16,713,922-16,693,294	19251	19278	(1381)	+	(AAT)n	Simple_repeat	1	26	(0)	306	
396	21.8	10.9	9.1	scaffold42_16,713,922-16,693,294	19833	20080	(579)	+	MamRep38	DNA/hAT	1	252	(43)	307	
1990	21.1	4.7	0.5	scaffold42_16,713,922-16,693,294	20253	20653	(6)	C	LTR3_EC	LTR/ERVL	(8)	740	323	308	
449	30.0	0.6	1.8	scaffold52_8,175,534-8,196,159	1	173	(20453)	+	L2a	LINE/L2	2445	2615	(804)	309	
1140	10.8	15.3	0.9	scaffold52_8,175,534-8,196,159	181	376	(20250)	C	SINEC1B_AMe	SINE/tRNA	(4)	224	1	310	
424	30.4	6.8	0.0	scaffold52_8,175,534-8,196,159	380	540	(20086)	+	L2a	LINE/L2	2605	2776	(643)	309	
405	26.2	6.0	0.9	scaffold52_8,175,534-8,196,159	584	813	(19813)	+	L2a	LINE/L2	2868	3162	(264)	309	
399	17.5	2.4	2.4	scaffold52_8,175,534-8,196,159	814	895	(19731)	C	MER34C_EC	LTR/ERV1	(20)	582	501	311	
651	20.3	0.8	0.8	scaffold52_8,175,534-8,196,159	912	1035	(19591)	+	SINEC2_AMe	SINE/tRNA	1	124	(9)	312	
302	13.0	14.5	0.5	scaffold52_8,175,534-8,196,159	1036	1124	(19502)	+	SINEC_c2	SINE/tRNA	125	225	(0)	313	*
1197	20.2	13.0	0.3	scaffold52_8,175,534-8,196,159	1118	1453	(19173)	C	MER34C_EC	LTR/ERV1	(142)	379	1	311	
379	25.8	10.8	3.1	scaffold52_8,175,534-8,196,159	1454	1615	(19011)	+	L2a	LINE/L2	3161	3339	(87)	309	
33	0.0	0.0	0.0	scaffold52_8,175,534-8,196,159	1616	1646	(18980)	+	(T)n	Simple_repeat	1	31	(0)	314	
379	22.0	8.8	1.9	scaffold52_8,175,534-8,196,159	1647	1716	(18910)	+	L2a	LINE/L2	3311	3388	(38)	309	
422	30.6	3.5	2.5	scaffold52_8,175,534-8,196,159	2286	2483	(18143)	+	L1ME4b	LINE/L1	5928	6127	(18)	315	
16	15.5	0.0	2.7	scaffold52_8,175,534-8,196,159	3915	3997	(16629)	+	(TCATTTC)n	Simple_repeat	1	80	(0)	316	
260	38.0	0.6	4.4	scaffold52_8,175,534-8,196,159	4788	4952	(15674)	+	MIR	SINE/MIR	53	211	(51)	317	
12	14.4	0.0	0.0	scaffold52_8,175,534-8,196,159	5589	5612	(15014)	+	(TAA)n	Simple_repeat	1	24	(0)	318	
445	25.5	0.0	0.9	scaffold52_8,175,534-8,196,159	6322	6432	(14194)	+	MIR	SINE/MIR	61	170	(92)	319	
207	16.7	0.0	0.0	scaffold52_8,175,534-8,196,159	6444	6479	(14147)	+	L2b	LINE/L2	3322	3357	(18)	320	*
218	17.5	0.0	2.5	scaffold52_8,175,534-8,196,159	6450	6490	(14136)	+	MIRb	SINE/MIR	225	264	(4)	321	
183	16.1	0.0	0.0	scaffold52_8,175,534-8,196,159	7620	7650	(12976)	C	L2	LINE/L2	(1036)	2383	2353	322	
244	31.9	10.1	3.3	scaffold52_8,175,534-8,196,159	7655	7881	(12745)	C	MIR	SINE/MIR	(13)	249	8	323	
396	33.8	5.6	2.6	scaffold52_8,175,534-8,196,159	8302	8605	(12021)	C	L2	LINE/L2	(1368)	2051	1739	322	
314	32.4	2.5	5.3	scaffold52_8,175,534-8,196,159	8861	9066	(11560)	C	MIRb	SINE/MIR	(26)	242	45	324	
14	0.0	0.0	0.0	scaffold52_8,175,534-8,196,159	9274	9291	(11335)	+	(GTT)n	Simple_repeat	1	18	(0)	325	
446	23.2	4.3	5.4	scaffold52_8,175,534-8,196,159	9988	10196	(10430)	C	LTRIS4A	LTR/ERV1	(42)	432	205	326	
194	30.4	11.1	0.7	scaffold52_8,175,534-8,196,159	11714	11839	(8787)	+	L2c	LINE/L2	3249	3387	(0)	327	
19	14.5	0.0	0.0	scaffold52_8,175,534-8,196,159	11944	11974	(8652)	+	(T)n	Simple_repeat	1	31	(0)	328	
18	4.3	0.0	4.0	scaffold52_8,175,534-8,196,159	12537	12562	(8064)	+	(CA)n	Simple_repeat	1	25	(0)	329	
712	26.4	7.6	0.8	scaffold52_8,175,534-8,196,159	13041	13277	(7349)	C	MIR	SINE/MIR	(1)	261	9	330	
257	30.0	3.3	0.0	scaffold52_8,175,534-8,196,159	13717	13806	(6820)	+	L2c	LINE/L2	3292	3384	(3)	331	
1582	21.1	6.4	4.3	scaffold52_8,175,534-8,196,159	14339	14837	(5789)	C	MER34A	LTR/ERV1	(6)	501	2	332	
855	13.0	17.2	1.7	scaffold52_8,175,534-8,196,159	15022	15178	(5448)	C	SINEC_b1	SINE/tRNA	(11)	183	3	333	
189	15.8	2.6	0.0	scaffold52_8,175,534-8,196,159	17561	17598	(3028)	C	MIR3	SINE/MIR	(87)	121	83	334	
12	15.8	0.0	3.5	scaffold52_8,175,534-8,196,159	18154	18183	(2443)	+	(TGAT)n	Simple_repeat	1	29	(0)	335	
184	26.1	8.7	0.0	scaffold52_8,175,534-8,196,159	18374	18442	(2184)	+	L2b	LINE/L2	3297	3371	(4)	336	
589	13.6	0.9	1.9	scaffold52_8,175,534-8,196,159	19084	19188	(1438)	C	MLT1D	LTR/ERVL-MaLR	(0)	505	402	337	
334	35.2	6.2	7.5	scaffold52_8,175,534-8,196,159	19228	19700	(926)	+	L2b	LINE/L2	2907	3372	(3)	338	
230	14.7	0.0	0.0	scaffold5_13,107,950-13,128,518	200	233	(20336)	+	PlatSat2A	Satellite	263	296	(737)	339	
510	17.2	4.2	1.6	scaffold5_13,107,950-13,128,518	640	757	(19812)	+	SINEC2_AMe	SINE/tRNA	2	122	(11)	340	
14	8.8	0.0	0.0	scaffold5_13,107,950-13,128,518	805	828	(19741)	+	(AATT)n	Simple_repeat	1	24	(0)	341	
245	33.6	6.2	0.0	scaffold5_13,107,950-13,128,518	1049	1161	(19408)	+	MIRb	SINE/MIR	41	160	(108)	342	
440	28.2	7.8	3.6	scaffold5_13,107,950-13,128,518	1175	1417	(19152)	C	MamRep1527	LTR	(466)	503	251	343	
2305	18.3	9.8	0.0	scaffold5_13,107,950-13,128,518	2220	2677	(17892)	C	MLT1D	LTR/ERVL-MaLR	(0)	505	3	344	
187	29.6	0.0	0.0	scaffold5_13,107,950-13,128,518	2764	2817	(17752)	C	L2c	LINE/L2	(37)	3350	3297	345	
494	28.5	9.4	0.9	scaffold5_13,107,950-13,128,518	2843	3044	(17525)	C	MIR	SINE/MIR	(18)	244	26	346	
15	33.0	0.0	0.0	scaffold5_13,107,950-13,128,518	3170	3221	(17348)	+	(ATG)n	Simple_repeat	1	52	(0)	347	
27	0.0	0.0	0.0	scaffold5_13,107,950-13,128,518	4694	4719	(15850)	+	(TTAT)n	Simple_repeat	1	26	(0)	348	
1114	8.0	0.7	0.0	scaffold5_13,107,950-13,128,518	4728	4877	(15692)	C	L1-1_AMe	LINE/L1	(18)	6829	6679	349	
1506	22.4	7.2	7.0	scaffold5_13,107,950-13,128,518	4939	5437	(15132)	C	MLT1D	LTR/ERVL-MaLR	(3)	502	3	350	
234	20.3	3.3	3.3	scaffold5_13,107,950-13,128,518	5538	5598	(14971)	+	L3	LINE/CR1	3413	3473	(626)	351	
205	28.4	5.6	7.0	scaffold5_13,107,950-13,128,518	5659	5802	(14767)	C	MIRb	SINE/MIR	(30)	238	97	352	
16	3.2	8.8	2.8	scaffold5_13,107,950-13,128,518	5961	5994	(14575)	+	(AAC)n	Simple_repeat	1	36	(0)	353	
941	28.2	5.7	2.9	scaffold5_13,107,950-13,128,518	6204	6605	(13964)	+	L2	LINE/L2	1597	2009	(1410)	354	
387	27.3	1.1	0.0	scaffold5_13,107,950-13,128,518	6606	6693	(13876)	C	MER94	DNA/hAT-Blackjack	(0)	134	46	355	
628	29.6	12.1	4.1	scaffold5_13,107,950-13,128,518	6733	7138	(13431)	C	LTR16	LTR/ERVL	(1)	437	1	356	
533	32.2	2.1	0.4	scaffold5_13,107,950-13,128,518	7171	7404	(13165)	+	L2	LINE/L2	2012	2249	(1170)	354	
822	22.6	4.8	0.9	scaffold5_13,107,950-13,128,518	7432	7641	(12928)	+	MER58A	DNA/hAT-Charlie	5	222	(2)	357	
388	28.5	9.1	3.2	scaffold5_13,107,950-13,128,518	7679	7804	(12765)	+	L2a	LINE/L2	3107	3250	(176)	358	
2164	18.4	0.9	6.8	scaffold5_13,107,950-13,128,518	7805	8242	(12327)	C	MLT2D	LTR/ERVL	(0)	414	1	359	
388	27.6	8.8	4.4	scaffold5_13,107,950-13,128,518	8243	8413	(12156)	+	L2a	LINE/L2	3251	3426	(0)	358	
383	27.6	7.3	0.6	scaffold5_13,107,950-13,128,518	8412	8575	(11994)	+	L2	LINE/L2	2305	2479	(940)	354	*
742	22.8	6.2	1.4	scaffold5_13,107,950-13,128,518	8767	8975	(11594)	+	LTR16B1	LTR/ERVL	191	409	(71)	360	
451	23.4	4.6	3.2	scaffold5_13,107,950-13,128,518	10355	10560	(10009)	+	LTRIS4	LTR/ERV1	205	465	(42)	361	
947	15.0	11.2	2.7	scaffold5_13,107,950-13,128,518	11982	12186	(8383)	+	SINEC1B_AMe	SINE/tRNA	1	222	(6)	362	
255	8.8	0.0	0.0	scaffold5_13,107,950-13,128,518	12713	12746	(7823)	+	MARE8	DNA	148	181	(119)	363	
311	31.2	2.8	5.8	scaffold5_13,107,950-13,128,518	13070	13246	(7323)	C	MER5A	DNA/hAT-Charlie	(0)	189	18	364	
728	26.4	4.3	5.3	scaffold5_13,107,950-13,128,518	15175	15536	(5033)	+	LTR37B	LTR/ERV1	111	451	(17)	365	
13	3.9	7.1	0.0	scaffold5_13,107,950-13,128,518	15647	15658	(4911)	+	(TAAAA)n	Simple_repeat	1	14	(0)	366	
916	10.7	14.5	2.1	scaffold5_13,107,950-13,128,518	15659	15830	(4739)	+	SINEC_b1	SINE/tRNA	1	193	(1)	367	
13	3.9	7.1	0.0	scaffold5_13,107,950-13,128,518	15831	15845	(4724)	+	(TAAAA)n	Simple_repeat	5	19	(0)	368	
53	6.2	1.4	0.0	scaffold5_13,107,950-13,128,518	16721	16789	(3780)	+	(TCC)n	Simple_repeat	1	70	(0)	369	
534	24.6	9.7	1.5	scaffold5_13,107,950-13,128,518	18047	18348	(2221)	+	MLT1D	LTR/ERVL-MaLR	21	384	(121)	370	
881	24.9	4.1	5.9	scaffold5_13,107,950-13,128,518	18349	18642	(1927)	C	CarLTR9	LTR/ERV1	(1)	562	274	371	
450	22.4	1.8	2.8	scaffold5_13,107,950-13,128,518	18750	18859	(1710)	+	MLT1D	LTR/ERVL-MaLR	353	461	(0)	370	
1082	15.6	18.5	2.1	scaffold5_13,107,950-13,128,518	18964	19424	(1145)	+	MER34C_CF	LTR/ERV1	2	544	(6)	372	
2966	19.9	4.1	1.3	scaffold5_13,107,950-13,128,518	19850	20494	(75)	C	LTR21B1_EC	LTR/ERVL	(7)	861	190	373	
470	17.3	1.3	0.0	scaffold5_13,107,950-13,128,518	20495	20569	(0)	+	SINEC_c1	SINE/tRNA	1	76	(133)	374	
14	9.4	8.8	0.0	scaffold7_30,636,998-30,609,301	2112	2148	(25550)	+	(TTGTCTT)n	Simple_repeat	1	40	(0)	375	
663	12.6	0.0	0.0	scaffold7_30,636,998-30,609,301	2576	2670	(25028)	C	SINEC_old	SINE/tRNA	(17)	95	1	376	
285	15.6	17.2	0.0	scaffold7_30,636,998-30,609,301	3505	3568	(24130)	+	MER5C1	DNA/hAT-Charlie	59	133	(130)	377	*
378	21.1	2.6	9.3	scaffold7_30,636,998-30,609,301	3540	3654	(24044)	+	Charlie10	DNA/hAT-Charlie	2708	2815	(7)	378	
284	23.5	4.7	1.1	scaffold7_30,636,998-30,609,301	3666	3751	(23947)	+	L2b	LINE/L2	3287	3375	(0)	379	
266	34.0	5.1	1.0	scaffold7_30,636,998-30,609,301	3754	3851	(23847)	+	MIR	SINE/MIR	11	112	(150)	380	
507	27.7	10.8	0.6	scaffold7_30,636,998-30,609,301	4289	4575	(23123)	+	L1ME4b	LINE/L1	839	1154	(5967)	381	
674	23.8	11.3	5.2	scaffold7_30,636,998-30,609,301	4650	4897	(22801)	+	L1ME4b	LINE/L1	1300	1565	(5556)	381	
721	16.6	10.5	5.0	scaffold7_30,636,998-30,609,301	4898	5087	(22611)	+	SINEC1_AMe	SINE/tRNA	1	200	(1)	382	
674	20.0	35.5	2.0	scaffold7_30,636,998-30,609,301	5088	5215	(22483)	+	L1ME4b	LINE/L1	1566	1733	(5388)	381	
545	23.7	13.8	5.5	scaffold7_30,636,998-30,609,301	5227	5480	(22218)	+	L1ME4b	LINE/L1	5812	6085	(60)	383	
712	19.3	21.9	0.4	scaffold7_30,636,998-30,609,301	5482	5668	(22030)	C	SINEC1B_AMe	SINE/tRNA	(1)	227	1	384	
369	21.5	5.4	0.0	scaffold7_30,636,998-30,609,301	5867	5959	(21739)	+	L1ME4b	LINE/L1	1897	1994	(5127)	381	
879	26.9	8.5	2.4	scaffold7_30,636,998-30,609,301	6234	6622	(21076)	+	L1ME4b	LINE/L1	4883	5294	(852)	381	
1709	16.5	6.6	1.3	scaffold7_30,636,998-30,609,301	7121	7483	(20215)	C	L1_Carn5	LINE/L1	(0)	6509	6128	385	
481	23.0	6.2	0.0	scaffold7_30,636,998-30,609,301	7578	7690	(20008)	+	SINEC2_AMe	SINE/tRNA	1	120	(13)	386	
277	35.2	4.1	1.6	scaffold7_30,636,998-30,609,301	7924	8168	(19530)	+	L1MC	LINE/L1	4906	5156	(990)	387	
269	18.8	6.2	0.0	scaffold7_30,636,998-30,609,301	8347	8410	(19288)	+	L1ME4b	LINE/L1	5409	5476	(669)	381	
235	19.3	17.5	0.0	scaffold7_30,636,998-30,609,301	8411	8467	(19231)	+	MamRep38	DNA/hAT	2	68	(227)	388	*
364	26.7	8.8	3.9	scaffold7_30,636,998-30,609,301	8456	8603	(19095)	+	MamRep38	DNA/hAT	81	235	(60)	389	
479	31.4	6.3	0.9	scaffold7_30,636,998-30,609,301	8750	9051	(18647)	+	L1ME4b	LINE/L1	5670	5987	(158)	381	
736	16.6	9.0	4.1	scaffold7_30,636,998-30,609,301	9192	9379	(18319)	C	SINEC1_AMe	SINE/tRNA	(4)	197	1	390	
435	16.2	0.0	2.7	scaffold7_30,636,998-30,609,301	9416	9491	(18207)	+	tRNA-Lys-AAG	tRNA	1	74	(2)	391	
449	24.3	7.9	0.0	scaffold7_30,636,998-30,609,301	9600	9739	(17959)	+	L2a	LINE/L2	2452	2602	(817)	392	
534	26.9	7.5	1.4	scaffold7_30,636,998-30,609,301	9803	10002	(17696)	+	L2a	LINE/L2	2766	2977	(442)	392	
514	18.0	7.7	4.0	scaffold7_30,636,998-30,609,301	10430	10575	(17123)	+	LTRIS4	LTR/ERV1	242	468	(39)	393	

274	13.0	1.5	9.5	scaffold7_30,636,998-30,609,301	10612	10679	(17019)	+	KORV_I-int	LTR/ERV1	1	63	(7500)	394	
745	30.9	0.7	1.7	scaffold7_30,636,998-30,609,301	11111	11400	(16298)	+	KORV_I-int	LTR/ERV1	457	743	(6681)	394	
6801	29.8	3.3	3.6	scaffold7_30,636,998-30,609,301	11653	16298	(11400)	+	KORV_I-int	LTR/ERV1	1024	5682	(1742)	394	
360	35.2	0.0	0.0	scaffold7_30,636,998-30,609,301	16357	16498	(11200)	+	KORV_I-int	LTR/ERV1	5813	5954	(1470)	394	
291	28.4	0.0	0.0	scaffold7_30,636,998-30,609,301	16537	16610	(11088)	+	KORV_I-int	LTR/ERV1	6023	6096	(1328)	394	
891	33.4	3.0	2.6	scaffold7_30,636,998-30,609,301	16658	17118	(10580)	+	KORV_I-int	LTR/ERV1	6962	7424	(0)	394	
491	21.7	5.1	2.5	scaffold7_30,636,998-30,609,301	17532	17687	(10011)	+	LTRIS4	LTR/ERV1	309	468	(39)	395	
470	28.4	5.7	4.4	scaffold7_30,636,998-30,609,301	17751	17997	(9701)	+	L2a	LINE/L2	3075	3324	(102)	392	
239	28.1	0.0	0.0	scaffold7_30,636,998-30,609,301	18017	18080	(9618)	+	L2B_ME	LINE/L2	3383	3446	(1)	396	
878	24.2	7.3	2.4	scaffold7_30,636,998-30,609,301	19798	20110	(7588)	C	L1ME4a	LINE/L1	(4)	6120	5793	397	
589	42.8	7.4	0.5	scaffold7_30,636,998-30,609,301	20154	20355	(7343)	+	SINEC1B_AMe	SINE/tRNA	1	216	(12)	398	
677	11.7	1.9	1.9	scaffold7_30,636,998-30,609,301	20592	20696	(7002)	+	SINEC_old	SINE/tRNA	1	105	(7)	399	
421	27.3	1.6	0.8	scaffold7_30,636,998-30,609,301	20699	20820	(6878)	+	SINEC2_AMe	SINE/tRNA	2	124	(9)	400	
23	13.1	0.0	2.3	scaffold7_30,636,998-30,609,301	20851	20894	(6804)	+	A-rich	Low_complexity	1	43	(0)	401	
338	22.9	2.9	0.0	scaffold7_30,636,998-30,609,301	20981	21050	(6648)	+	tRNA-Lys-AAG	tRNA	2	73	(3)	402	
15	15.8	0.0	2.8	scaffold7_30,636,998-30,609,301	21062	21098	(6600)	+	A-rich	Low_complexity	1	36	(0)	403	
691	18.8	13.1	4.0	scaffold7_30,636,998-30,609,301	21143	21325	(6373)	C	SINEC1_AMe	SINE/tRNA	(2)	199	1	404	
315	21.8	10.8	0.9	scaffold7_30,636,998-30,609,301	21326	21439	(6259)	+	L1ME4c	LINE/L1	5459	5582	(501)	405	
421	13.6	0.0	0.0	scaffold7_30,636,998-30,609,301	21525	21590	(6108)	C	CarLTR1-int	LTR/ERV1	(517)	6098	6033	406	
1638	21.8	9.4	0.0	scaffold7_30,636,998-30,609,301	21736	22350	(5348)	C	CarERV2-int	LTR/ERV1	(1380)	6232	5560	407	
4729	20.8	2.5	1.2	scaffold7_30,636,998-30,609,301	22361	23325	(4373)	C	CarERV2-int	LTR/ERV1	(2702)	4910	3934	407	
284	32.8	6.7	0.8	scaffold7_30,636,998-30,609,301	23327	23446	(4252)	C	CarERV2-int	LTR/ERV1	(3927)	3497	3371	407	
2821	13.7	5.5	0.8	scaffold7_30,636,998-30,609,301	23448	23904	(3794)	C	CarERV2-int	LTR/ERV1	(3809)	3803	3326	408	
226	37.9	4.9	0.4	scaffold7_30,636,998-30,609,301	23905	24169	(3529)	C	CarERV2-int	LTR/ERV1	(4534)	2890	2614	408	
3844	18.4	3.6	0.4	scaffold7_30,636,998-30,609,301	24170	25154	(2544)	C	CarERV2-int	LTR/ERV1	(4560)	3052	2008	409	
672	12.3	0.0	1.9	scaffold7_30,636,998-30,609,301	25158	25265	(2433)	+	SINEC_old	SINE/tRNA	7	112	(0)	410	
2306	22.1	6.4	2.3	scaffold7_30,636,998-30,609,301	25271	25626	(2072)	C	CarERV2-int	LTR/ERV1	(5599)	2013	1556	409	
42	0.0	0.0	0.0	scaffold7_30,636,998-30,609,301	25627	25662	(2036)	+	(AC)n	Simple_repeat	1	36	(0)	411	
2306	23.0	5.3	1.7	scaffold7_30,636,998-30,609,301	25663	26010	(1688)	C	CarERV2-int	LTR/ERV1	(5959)	1653	1290	409	*
4009	16.6	5.0	3.2	scaffold7_30,636,998-30,609,301	25999	27328	(370)	C	CarERV2-int	LTR/ERV1	(6233)	1379	1	409	
1324	22.9	2.9	2.5	scaffold7_30,636,998-30,609,301	27329	27677	(21)	C	CarLTR1B2	LTR/ERV1	(4)	542	144	409	
458	16.9	16.8	0.7	scaffold80_7,084,287-7,112,941	4	122	(28533)	C	MLT1C	LTR/ERVL-MaLR	(59)	408	271	412	
15	9.8	0.0	0.0	scaffold80_7,084,287-7,112,941	210	231	(28424)	+	(AT)n	Simple_repeat	1	22	(0)	413	
20	0.0	0.0	0.0	scaffold80_7,084,287-7,112,941	233	254	(28401)	+	(GA)n	Simple_repeat	1	22	(0)	414	
16	0.0	0.0	0.0	scaffold80_7,084,287-7,112,941	333	350	(28305)	+	(A)n	Simple_repeat	1	18	(0)	415	
389	16.9	0.0	3.4	scaffold80_7,084,287-7,112,941	419	510	(28145)	+	L1-1_AMe	LINE/L1	6751	6839	(8)	416	
532	26.8	3.9	0.5	scaffold80_7,084,287-7,112,941	511	690	(27965)	C	MER20B	DNA/hAT-Charlie	(401)	382	197	417	
234	11.6	0.0	2.3	scaffold80_7,084,287-7,112,941	705	748	(27907)	C	MER20B	DNA/hAT-Charlie	(702)	81	39	417	
201	36.4	10.1	0.0	scaffold80_7,084,287-7,112,941	1345	1443	(27212)	+	L3	LINE/CR1	3562	3670	(429)	418	
3152	16.3	6.0	3.2	scaffold80_7,084,287-7,112,941	1628	1694	(26961)	C	L1MB1	LINE/L1	(5)	6163	6111	419	
1597	7.5	0.0	0.0	scaffold80_7,084,287-7,112,941	1695	1894	(26761)	+	SINEC1_AMe	SINE/tRNA	1	200	(1)	420	
3152	16.3	6.0	3.2	scaffold80_7,084,287-7,112,941	1895	2471	(26184)	C	L1MB1	LINE/L1	(58)	6110	5501	419	
21	23.9	1.7	0.0	scaffold80_7,084,287-7,112,941	2480	2538	(26117)	+	(TATA)n	Simple_repeat	1	60	(0)	421	
499	23.9	2.4	0.4	scaffold80_7,084,287-7,112,941	2540	2684	(25971)	C	L1MB1	LINE/L1	(1368)	4977	4814	419	
333	33.7	1.7	1.1	scaffold80_7,084,287-7,112,941	2685	2858	(25797)	C	Sat-1_TSy	Satellite	(1280)	1956	1782	422	
1063	16.0	2.8	2.3	scaffold80_7,084,287-7,112,941	2859	2885	(25770)	C	L1MB1	LINE/L1	(1503)	4637	4611	419	
60	0.0	3.3	0.0	scaffold80_7,084,287-7,112,941	2886	2946	(25709)	+	(AAAG)n	Simple_repeat	1	63	(0)	423	
1063	16.0	2.8	2.3	scaffold80_7,084,287-7,112,941	2947	3136	(25519)	C	L1MB1	LINE/L1	(1530)	4610	4419	419	
378	29.4	4.2	3.6	scaffold80_7,084,287-7,112,941	3581	3746	(24909)	C	Tigger15a	DNA/TcMar-Tigger	(416)	299	133	424	
47	27.0	1.6	4.4	scaffold80_7,084,287-7,112,941	4771	4955	(23700)	+	(TTTC)n	Simple_repeat	1	180	(0)	425	
15	26.9	3.3	1.6	scaffold80_7,084,287-7,112,941	5532	5592	(23063)	+	(ATGATA)n	Simple_repeat	1	62	(0)	426	
70	13.6	0.0	2.4	scaffold80_7,084,287-7,112,941	5671	5798	(22857)	+	(TATATA)n	Simple_repeat	1	125	(0)	427	
34	0.0	0.0	0.0	scaffold80_7,084,287-7,112,941	5802	5830	(22825)	+	(TG)n	Simple_repeat	1	29	(0)	428	
257	34.2	0.8	1.7	scaffold80_7,084,287-7,112,941	5841	5959	(22696)	+	MER5A	DNA/hAT-Charlie	72	189	(0)	429	
555	33.0	2.3	0.5	scaffold80_7,084,287-7,112,941	7017	7235	(21420)	+	MIRb	SINE/MIR	30	252	(16)	430	
346	29.9	12.1	1.7	scaffold80_7,084,287-7,112,941	7707	8047	(20608)	C	L2b	LINE/L2	(0)	3426	3055	431	
667	22.3	2.9	8.0	scaffold80_7,084,287-7,112,941	8126	8335	(20320)	+	SINEC1_AMe	SINE/tRNA	1	200	(1)	432	
17	19.5	0.0	0.0	scaffold80_7,084,287-7,112,941	8337	8367	(20288)	+	A-rich	Low_complexity	1	31	(0)	433	
379	22.7	6.1	4.3	scaffold80_7,084,287-7,112,941	10071	10185	(18470)	+	MIRb	SINE/MIR	20	136	(132)	434	
16	21.8	2.6	0.0	scaffold80_7,084,287-7,112,941	11842	11879	(16776)	+	(TA)n	Simple_repeat	1	39	(0)	435	
522	21.4	4.1	4.0	scaffold80_7,084,287-7,112,941	12014	12219	(16436)	C	LTRIS4	LTR/ERV1	(42)	465	205	436	
353	26.1	7.7	2.1	scaffold80_7,084,287-7,112,941	12960	13129	(15526)	+	Mon1g0	SINE/MIR	1	179	(115)	437	
239	24.7	5.7	3.3	scaffold80_7,084,287-7,112,941	14690	14777	(13878)	+	MIR3	SINE/MIR	15	104	(104)	438	
541	23.4	7.6	5.3	scaffold80_7,084,287-7,112,941	14912	15096	(13559)	+	MER5A	DNA/hAT-Charlie	1	189	(0)	439	
190	33.3	6.4	1.0	scaffold80_7,084,287-7,112,941	15225	15318	(13337)	+	L2b	LINE/L2	3264	3362	(13)	440	
285	31.1	2.4	2.4	scaffold80_7,084,287-7,112,941	15330	15454	(13201)	+	MIRb	SINE/MIR	86	210	(58)	441	
45	6.0	0.0	0.0	scaffold80_7,084,287-7,112,941	15483	15534	(13121)	+	(GT)n	Simple_repeat	1	52	(0)	442	
400	37.2	7.7	3.0	scaffold80_7,084,287-7,112,941	15622	16037	(12618)	C	MamGypLTR1c	LTR/Gypsy	(119)	684	250	443	
293	29.5	14.3	0.0	scaffold80_7,084,287-7,112,941	16158	16262	(12393)	C	MamGypLTR1c	LTR/Gypsy	(620)	193	74	443	
678	27.3	1.4	2.3	scaffold80_7,084,287-7,112,941	16297	16517	(12138)	+	MER20	DNA/hAT-Charlie	1	219	(0)	444	
456	22.4	1.0	0.0	scaffold80_7,084,287-7,112,941	17181	17278	(11377)	C	MER45C	DNA/hAT-Tip100	(11)	942	844	445	
816	9.4	24.5	4.3	scaffold80_7,084,287-7,112,941	17279	17433	(11222)	+	SINEC_b1	SINE/tRNA	1	185	(9)	446	
39	4.1	3.4	8.2	scaffold80_7,084,287-7,112,941	17438	17526	(11129)	+	(TA)n	Simple_repeat	1	85	(0)	447	
39	6.8	0.0	0.0	scaffold80_7,084,287-7,112,941	17527	17573	(11082)	+	(TG)n	Simple_repeat	1	47	(0)	448	
292	19.5	0.0	3.9	scaffold80_7,084,287-7,112,941	17588	17667	(10988)	C	MER45C	DNA/hAT-Tip100	(97)	856	780	445	
1933	23.8	2.0	3.3	scaffold80_7,084,287-7,112,941	18914	19460	(9195)	+	Arthur2	DNA/hAT-Tip100	442	981	(2719)	449	
13	22.2	3.5	5.3	scaffold80_7,084,287-7,112,941	19894	19951	(8704)	+	(ATT)n	Simple_repeat	1	57	(0)	450	
1367	21.2	10.3	0.6	scaffold80_7,084,287-7,112,941	20099	20492	(8163)	C	L1MA7	LINE/L1	(0)	6894	6510	451	
75	13.4	5.4	0.0	scaffold80_7,084,287-7,112,941	20862	21008	(7647)	+	(AT)n	Simple_repeat	1	155	(0)	452	
17	0.0	0.0	0.0	scaffold80_7,084,287-7,112,941	21198	21216	(7439)	+	(TG)n	Simple_repeat	1	19	(0)	453	
16	0.0	5.0	0.0	scaffold80_7,084,287-7,112,941	21216	21235	(7420)	+	(TA)n	Simple_repeat	1	21	(0)	454	*
463	29.6	8.6	0.6	scaffold80_7,084,287-7,112,941	21332	21494	(7161)	C	MLT1H	LTR/ERVL-MaLR	(0)	549	374	455	
266	37.1	1.0	7.2	scaffold80_7,084,287-7,112,941	21541	21746	(6909)	C	L2c	LINE/L2	(200)	3187	2994	456	
51	2.1	0.0	0.0	scaffold80_7,084,287-7,112,941	22417	22464	(6191)	+	(AC)n	Simple_repeat	1	48	(0)	457	
465	27.2	2.3	1.6	scaffold80_7,084,287-7,112,941	22742	22937	(5718)	+	L1MB7	LINE/L1	5983	6183	(1)	458	
231	35.6	3.3	0.0	scaffold80_7,084,287-7,112,941	23168	23257	(5398)	C	L2a	LINE/L2	(30)	3396	3304	459	
37	0.0	0.0	0.0	scaffold80_7,084,287-7,112,941	23468	23499	(5156)	+	(TG)n	Simple_repeat	1	32	(0)	460	
782	28.3	1.5	1.5	scaffold80_7,084,287-7,112,941	23891	24091	(4564)	C	MER20	DNA/hAT-Charlie	(3)	216	16	461	
459	28.2	2.3	0.0	scaffold80_7,084,287-7,112,941	24162	24292	(4363)	+	MER94	DNA/hAT-Blackjack	1	134	(0)	462	
1560	5.5	1.0	1.5	scaffold80_7,084,287-7,112,941	24351	24552	(4103)	C	SINEC1_AMe	SINE/tRNA	(0)	201	1	463	
233	29.2	16.1	1.5	scaffold80_7,084,287-7,112,941	25706	25823	(2832)	C	MIR3	SINE/MIR	(55)	153	19	464	
488	26.4	0.0	0.0	scaffold80_7,084,287-7,112,941	25897	26006	(2649)	+	MER81	DNA/hAT-Blackjack	5	114	(0)	465	
330	37.0	9.0	1.3	scaffold80_7,084,287-7,112,941	26162	26372	(2283)	+	MER115	DNA/hAT-Tip100	23	249	(444)	466	
1227	30.0	1.5	4.4	scaffold80_7,084,287-7,112,941	26392	26789	(1866)	+	MER115	DNA/hAT-Tip100	307	693	(0)	467	
1398	9.8	3.1	0.0	scaffold80_7,084,287-7,112,941	27049	27242	(1413)	C	SINEC1_AMe	SINE/tRNA	(1)	200	1	468	
207	35.1	1.1	1.1	scaffold80_7,084,287-7,112,941	27550	27644	(1011)	C	MIRb	SINE/MIR	(0)	268	174	469	
1651	7.7	3.2	0.0	scaffold80_7,084,287-7,112,941	27676	27895	(760)	+	L1_Canid_	LINE/L1	6223	6449	(2)	470	
188	24.5	2.0	2.0	scaffold80_7,084,287-7,112,941	28577	28626	(29)	C	MIRb	SINE/MIR	(120)	148	99	471	
